# Suppressing Effects of Docosahexaenoic Acid–Containing Diets on Oxidative Stress and Fibrosis in 5/6 Nephrectomized Rats

**DOI:** 10.34067/KID.0000000000000152

**Published:** 2023-05-24

**Authors:** Hiroki Muramatsu, Naoe Akimoto, Katsuhiko Yajima, Michio Hashimoto, Masanori Katakura

**Affiliations:** 1 Laboratory of Nutritional Physiology, Department of Pharmaceutical Sciences, Faculty of Pharmacy and Pharmaceutical Sciences, Josai University, Sakado, Japan; 2 Faculty of Medicine, Shimane University, Izumo, Japan

**Keywords:** CKD, basic science, chronic renal failure, inflammation, kidney fibrosis, oxidative stress

## Abstract

**Key Points:**

- Increased albuminuria on 5/6 nephrectomized rats, as reported earlier, is attenuated by arachidonic acid–containing and docosahexaenoic acid (DHA)–containing diets.
- This study established that DHA affects both oxidative stress and fibrosis in the kidney.
- DHA suppressed the oxidative stress and fibrosis, hence suppressing the progression of renal failure.

**Background:**

Urinary albumin excretion gradually increases after nephrectomy, which eventually progresses toward renal failure. Our previous study had reported that arachidonic acid (ARA)–containing or docosahexaenoic acid (DHA)–containing diet attenuates the increasing urinary albumin excretion. This study aimed to investigate the effects of ARA-containing or/and DHA-containing diets on oxidative stress and fibrosis that cause kidney injury in 5/6 nephrectomized rats.

**Methods:**

Sprague–Dawley rats were randomly divided into control group, ARA group, DHA group, and ARA+DHA group. Rats underwent 5/6 kidney removal and were fed ARA-containing or/and DHA-containing diet each five groups continuously for 4 weeks. We collected urine, plasma, and kidney samples 4 weeks after surgery and investigated the effects of ARA-containing and DHA-containing diets on oxidative stress, inflammation, and fibrosis in the kidney.

**Results:**

Urinary albumin excretion, indoxyl sulfate, reactive oxygen species, TNF-*α* levels, and fibrosis in the kidney were all increased on nephrectomy; however, they were attenuated after feeding the rats with DHA-containing diet.

**Conclusion:**

One possible mechanism of preventing chronic renal failure would be the suppression of indoxyl sulfate accumulation, oxidative stress, and kidney fibrosis arising due to nephrectomy. The results collectively suggested that DHA-containing diets can suppress the progression of renal failure.

## Graphical abstract

**Figure:**
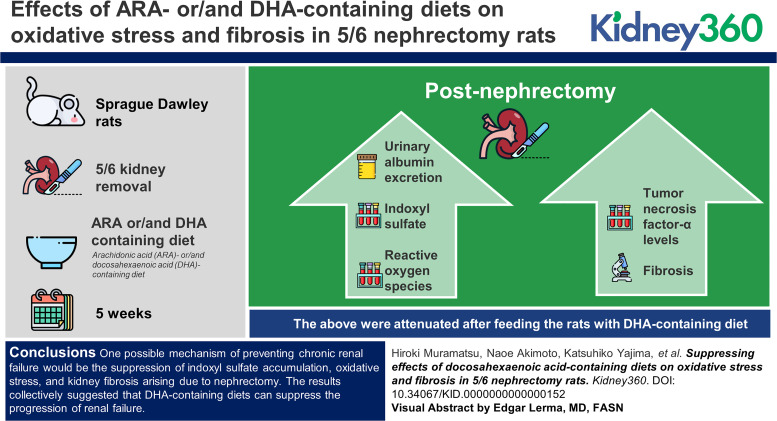

## Introduction

Arachidonic acids (ARAs), which are omega-6 (*ω*-6) polyunsaturated fatty acids (PUFAs), are precursors of a wide range of metabolites. ARA metabolites are known to be involved in the promotion or resolution of inflammation. Prostaglandins are produced from ARA by enzymes, such as cyclooxygenase (COX) and lipoxygenase (LOX). Prostaglandins are involved in the development and maintenance of normal renal function by adjusting the blood flow to kidneys, the amount of body water, and the balance of sodium concentration.^1^ Thromboxane B2 (TXB2) and leukotriene A4, also produced from ARA by the action of COX and LOX, have been reported to be involved in the promotion of inflammation. Epoxyeicosatrienoic acids are produced from ARA by the action of cytochrome P450 epoxygenase and exhibit reno-protective effects through vasodilation, antihypertension, antiapoptosis, and anti-inflammation.^2^ Docosahexaenoic acid (DHA) and eicosapentaenoic acid (EPA) are *ω*-3 PUFAs that are involved in anti-inflammatory processes.^3^ Protectin D and resolvin E, both DHA metabolites, and 18-hydroxy eicosapentaenoic acid (HEPE), an EPA metabolite, are produced by enzymes, such as COX, LOX, and CYP450. Protectin D and resolvin E protect against ischemic acute renal failure,^4^ IgA-induced or cyclosporine A-induced nephrotoxicity,^5–9^ streptozocin-induced type 1 diabetes,^10^ and type 2 diabetic nephropathy in rodents.^11–14^

Overproduction of reactive oxygen species (ROS) has been reported to occur due to oxidative stress, leading to the progression of CKD.^15,16^ Oxidative stress in the kidney increases kidney fibrosis, glomerulosclerosis, and proteinuria by promoting the production of TNF-*α* and TGF-*β*_1_.^16^

In our previous study, intake of ARA-containing and DHA-containing diet was shown to attenuate the lipid peroxide (LPO) levels in plasma 4 weeks after nephrectomy, and it was important for the suppression of renal function at 16 weeks after nephrectomy.^17^ However, how the feeding of 5/6 nephrectomized rats with ARA-containing and DHA-containing diets affected the oxidative stress and fibrosis in the kidney at 4 weeks after nephrectomy still remains unclear. This study aimed to assess the effects of ARA-containing and DHA-containing diets on oxidative stress and fibrosis in the kidneys of 5/6 nephrectomized rats and to examine the association of PUFA metabolites with kidney and renal function at 4 weeks after nephrectomy.

## Methods

### Animals and Diets

All experiments were performed in accordance with the Guidelines for Animal Experimentation of Josai University in Japan and were approved by the Rat Care and Use Committee of the same institution (H31076). This study was performed in compliance with the Guiding Principles for the Care and Use of Animals in the Field of Physiological Sciences of the Physiological Society of Japan, and adhered to the NIH Guide for the Care and Use of Laboratory Animals. Male Sprague–Dawley (6 weeks old) rats were purchased from Sankyo Labo Service Corporation (Tokyo, Japan) and were housed in a room under controlled temperature (25°C±2°C), humidity (60%±5%), and light-dark cycle (7:00–19:00). Fatty acid composition of diet on the basis of AIN-76A, in which the adjusted ratio of *ω*-6 PUFA to *ω*-3 PUFA is 2 to 1 and the PUFA to SFA to MUFA ratio is 1 to 1 to 1. The fatty acid composition of the diets is presented in Table 1.

**Table 1:** Fatty acid composition of diets (%) on the basis of AIN-76A

Percentage	Control	ARA	DHA	ARA+DHA
PLA (16:0)	27.6	27.2	27.3	28.1
STA (18:0)	4.2	4.6	4.4	4.8
OLA (18:1)	31.5	29.8	29.9	28.8
LA (18:2*ω*-6)	22.3	17.7	21.7	16.4
ALA (18:3*ω*-3)	11.3	11.6	6.2	5.8
ARA (20:4*ω*-6)	0	4.1	0.2	4.2
EPA (20:5*ω*-3)	0	0	0.8	0.8
DHA (22:6*ω*-3)	0	0	4.0	4.0
SFA	33.1	34.2	33.4	35.6
MUFA	31.9	30.3	31.3	30.2
PUFA	34.0	34.6	33.8	32.7
*ω*-6/*ω*-3	1.98	1.91	2.01	2.01

ARA, arachidonic acid; DHA, docosahexaenoic acid; PLA, palmitic acid; STA, stearic acid; OLA, oleic acid; LA, linoleic acid; ALA, *α*-linoleinic acid; EPA, eicosapentaenoic acid; SFA, saturated fatty acid; MUFA, monounsaturated fatty acid; PUFA, polyunsaturated fatty acid; *ω*-6/*ω*-3, omega-6/omega-3.

### Nephrectomy

Rats for 5/6 nephrectomy were randomly assigned into four groups, one control group and three groups fed ARA-containing, DHA-containing, or ARA+DHA-containing diets along with water *ad libitum*, for 4 weeks. Then five sixths of the kidneys were removed from each rat. The rats were anesthetized using a mix of three anesthetic types: medetomidine/midazolam/butorphanol (0.5/5.0/2.5 mg/ml). First, two thirds of the left side kidney were removed, and then, after 2 weeks, the whole right side kidney was removed.

### Sample Collection

Rats were housed in individual metabolic cages (SN-781, Shinano, Saitama, Japan), and urine and feces were separately collected for 24 hours every 4 weeks. Blood samples were collected from the orbital sinus after anesthetizing the animals using an anesthesia apparatus (SN-487-IT, Shinano). Part of each blood sample was examined for creatinine levels after collecting the plasma by centrifugation. Urine samples were centrifuged at 950 *g* for 10 minutes at 4°C, and the top layer was collected. Part of each urine sample was used to measure urinary albumin and creatinine clearance (Ccr). Ccr was calculated as described previously. After 4 weeks, the rats were anesthetized using isoflurane, blood was collected from abdominal inferior vena cava, and the kidney was stored at −80°C.

### Analysis of Fatty Acid Composition

Fatty acid (FA) composition of plasma, red blood cell membrane, and kidney homogenates was determined using gas chromatography, as described previously.^17^ FA composition of kidneys was measured using the GC-2014 (Shimadzu, Kyoto, Japan) equipped with a flame ionization detector and an automatic sampler (AOC-20i, Shimadzu). GC was performed using a capillary column (DB-WAX 30 m×0.53 mm×I.D 3 *μ*m, Agilent Technologies, CA). For sample injection, the split method was used with a split ratio of 10.0; the carrier gas was nitrogen. GC was set up at 280°C with flame ionization detector, initially maintained at 100°C for 4 minutes in a column oven. The temperature rose to 200°C at 15°C/min and was maintained there for 5 minutes; thereafter, the temperature rose to 260°C at 2.5°C/min and was maintained there for 10 minutes. Run time per sample was set to 49.5 minutes.

### Analysis of Fatty Acid Metabolites and Indoxyl Sulfate in Kidney

For samples of fatty acid metabolites, kidney homogenates used in this study, and in a previous study,^17^ were prepared as described previously.^17^

To prepare sample for determining the indoxyl sulfate (IS) level in the kidney, acetonitrile was added to each kidney homogenate and the mixed sample was kept at −30°C for 30 minutes. The samples were centrifuged at 13,420 *g* for 10 minutes at 4°C, and the top layer was collected therefrom. The IS level in the kidneys was measured using LC/MS/MS.

### LC-MS-MS Analysis

The LC/MS/MS system consisted of LC-40D XS pumps (Shimadzu), a SIL-40C XS auto-sampler (Shimadzu), a CTO-40C column oven (Shimadzu), a SCL-40 system controller (Shimadzu), and a triple quadrupole mass spectrometer LCMS-8050 (Shimadzu). For analysis of fatty acid metabolites, a reverse phase column (Kinetex C8, 2.1×150 mm, 2.6 *µ*m, Phenomenex, CA) at 40°C was used for chromatographic separation. For mobile phases A and B, 0.1% formic acid in water and acetonitrile, respectively, in a 90:10 ratio were ramped up to 75:25 ratio after 5 minutes, to 65:35 ratio after another 5 minutes, to 25:75 ratio after 10 minutes, to 2:98 ratio after 8 minutes, to 90:10 ratio after 2 minutes, and held there for another 2 minutes with a flow rate of 0.4 ml/min. Sample injection volume was 5 *µ*l. MS-MS analyses were conducted in negative ion mode, and fatty acid metabolites were detected and quantified using multiple reaction monitoring (MRM). Conditions for the detection of each compound by MRM have been used (LC/MS/MS method package version 3. 225-24872B. January 2019, Shimadzu). Peaks were selected, and their areas were calculated using LabSolutions (Shimadzu).

To analyze the IS level in the kidney, a reverse phase column (ReproSil-Pur 120 C18-AQ, 3 *μ*m, 100×2 mm, Dr. Maisch, Ammerbuch, Germany) at 40°C was used for chromatographic separation. For mobile phases A and B, 0.1% formic acid and 20% acetonitrile in water and 10 mM ammonium acetate in 80% acetonitrile, respectively, in a 90:10 ratio for 2 minutes were ramped up to 0:100 ratio for 13 minutes, then to a 90:10 ratio for 3 minutes, and held a flow rate of 0.4 ml/min. Sample injection volume was 5 *µ*l. MS-MS analyses were conducted in negative ion mode, and each compound was detected and quantified using MRM. Conditions for the detection of each compound using MRM are presented in Supplemental Table 1. IS concentration was calculated using the standard curve of IS.

### Analysis of Oxidative Stress Status

ROS and peroxynitrate (ONOO^−^) levels were measured as described previously.^17^ To measure the LPO levels, 8.1% sodium dodecyl sulfate, 0.4% thiobarbituric acid/20% acetic acid, and purified water were added to the kidney homogenate, and the reaction was promoted by 1-hour incubation at 95°C. Samples were shaken for 5 minutes after the sequential addition of purified water and 1-butanol/pyridine. Thereafter, samples were centrifuged at 1860 *g* for 20 minutes at 20°C, and the top layer was collected. Absorbance of samples was measured using a spectrophotometer. The amount of LPO was calculated from the absorbance reading using a linear calibration curve of 1,1,3,3-tetramethoxypropane, taken as the internal standard.

### Analysis of mRNA Levels of an Antioxidant Enzyme

Total RNA was isolated using ISOGEN II (NIPPON GENE, Tokyo, Japan). cDNA was synthesized using a QuantiTect Reverse Transcription Kit (Qiagen, Hilden, Germany) and amplified using a 7500 real-time PCR system (Applied Biosystems, Foster City, CA). Real-time PCR was conducted using a QuantiTect SYBR Green PCR Kit (Qiagen). The primer sequences are listed in Supplemental Table 2. The PCR conditions were as follows: 95°C for 15 seconds, 63°C for 30 seconds, and 72°C for 30 seconds. Relative changes in gene expression levels were determined using the 2^−ΔΔ*C*t^ method.

### Histological Evaluation

Kidney tissues were processed into formalin-fixed paraffin-embedded tissue blocks. The paraffin-embedded tissue was cut into 3-*μ*m sections. The paraffin sections of kidney tissue were deparaffinized and stained with hematoxylin and eosin (H&E), periodic acid–Schiff (PAS), and Masson trichrome (MT), followed by observation under a fluorescence microscope (BZ-X700; Keyence, Osaka, Japan).

In H&E staining, the overall coronal section was evaluated for vacuolization of kidney tissue. PAS staining was used to evaluate glomerular hypertrophy; 10 glomeruli were randomly selected from each rat for histological evaluation. MT staining was performed to calculate the proportion of kidney fibrosis with respect to the total kidney area (%).

For immunohistochemical staining, the paraffin sections of kidney tissue were deparaffinized and processed for antigen retrieval and blocking. Antigen retrieval was accomplished by heating the slides in a water bath at 95°C in 10 mM citrate buffer, pH 6.0 for 1 hour. After incubation with 5% goat serum to block nonspecific binding, *α*-smooth muscle actin (*α*-SMA) was detected using rabbit polyclonal primary antibodies (1/500; GeneTex, Irvine, CA), CD86 was detected using mouse monoclonal primary antibodies (1/100; Abcam, Cambridge, United Kingdom), and CD163 was detected using rabbit monoclonal primary antibodies (1/500; Abcam). *α*-SMA, CD86, and CD163 were incubated overnight with the primary antibody at 4°C in Tris-buffered saline containing 0.1% TWEEN 20 and 1% bovine serum albumin (Fujifilm Wako Pure Chemical Corporation, Osaka, Japan). After washing, *α*-SMA and CD163 were incubated with anti-rabbit IgG peroxidase conjugate (1/500; EMD Millipore, MA), and CD86 was incubated with anti-mouse IgG peroxidase conjugate (1/500; EMD Millipore) for 1 hour at 20°C. The secondary antibodies used were anti-rabbit IgG-HRP (1/1000; Sigma-Aldrich, MO) and anti-mouse IgG-HRP (1/1000; Sigma). Finally, for counterstaining, we used hematoxylin. *α*-SMA was positive in myofibroblasts, CD86 was positive in M1 macrophage, and CD163 was positive in M2 macrophage. *α*-SMA, CD86, and CD163 immunohistochemical staining were performed to calculate positive cells in overall kidney area (%).

### Determination of Osteopontin Levels in Urine

Concentrations of urinary osteopontin were measured using the Bio-Plex system that combines the principle of a sandwich immunoassay with fluorescent bead-based Luminex technology (Bio-Rad, CA).

### Statistical Analyses

All data are expressed as the means±SEM. Data were analyzed with the one-way ANOVA and Tukey honestly significant difference test. Differences between groups were considered significant at *P* < 0.05. All statistical analyses were performed using JMP (JMP Pro for MAC 16.0.0, SAS institute Japan, Tokyo, Japan).

## Results

### DHA Attenuated Albuminuria and Kidney Injury at 4 Weeks after Nephrectomy

To assess the renal status of 5/6 nephrectomized rats at 4 weeks after nephrectomy, we investigated body weight, kidney weight, Ccr, and urinary albumin excretion before and 4 weeks after nephrectomy (Table 2).

**Table 2:** Changes in renal parameters after nephrectomy

Parameters	Weeks	Sham	Nephrectomy			
		Control	Control	ARA	DHA	ARA+DHA
Body weight (g)	0	360.5±7.4	377.6±10.1	351.5±7.6	358.9±5.4	362.0±8.5
	4	497.0±12.3	429.4±22.8	447.4±8.1	445.6±10.2	429.4±21.3
Kidney weight (g)	0	No data	No data	No data	No data	No data
	4	3.3±0.1	3.3±0.5	3.2±0.4	2.7±0.7	3.1±0.3
Creatinine clearance	0	8.2±0.4	8.5±0.7	6.8±0.7	9.2±0.4	8.4±0.8
(ml/min per kg body weight)	4	8.3±0.3^*^	2.1±0.4^*^	2.1±0.3^*^	3.2±0.4^*^	2.2±0.23^*^
Urinary albumin	0	0.9±0.2	1.2±0.4	0.9±0.4	0.5±0.1	3.9±2.1
(g/24 h per kg)	4	9.7±4.3^*^	267.8±82.6^*^	182.9±40.4^*^	100.0±36.8^*^	209.4±100.6^*^

Values are presented as the mean±SEM (*n*=5–7). ARA, arachidonic acid; DHA, docosahexaenoic acid.

* Significant differences were observed between the experimental group and the CS group, as determined by ANOVA and Tukey HSD tests (*P* < 0.01).

Body weights were significantly decreased due to nephrectomy. However, kidney weight was not significantly different across the five groups, namely control sham (CS), control, ARA, DHA, and ARA+DHA. Ccr significantly decreased due to nephrectomy but was not affected by different PUFA levels.

**Figure 1:**
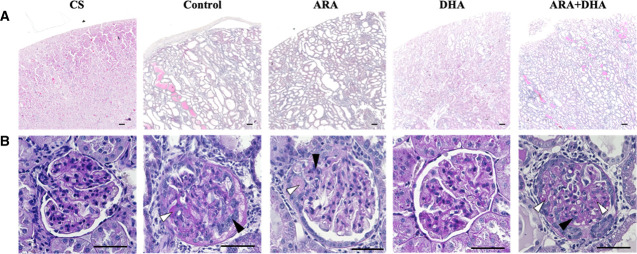
**DHA attenuated kidney injury at 4 weeks after nephrectomy.** Images of (A) H&E staining of kidney section and (B) PAS staining of glomeruli at 4 weeks after nephrectomy. Images of coronal sections of kidney and glomeruli from CS group, control group, ARA group, DHA group, and ARA+DHA group. Scale bar, 50 *µ*m. White arrow indicates glomerular hypertrophy. Black arrow indicates the increased number of mesangial cells. DHA, docosahexaenoic acid; H&E, hematoxylin and eosin; PAS, periodic acid–Schiff; CS, control sham; ARA, arachidonic acid.

In 5/6 nephrectomized rat model, glomerulosclerosis, inflammation, and fibrosis occur when GFR <50% compared to that in normal rat.^18,19^ In this study, the renal status of 5/6 nephrectomy rats indicated extremely severe renal conditions. Urinary albumin levels significantly increased due to nephrectomy and were attenuated 63% in the DHA group than in the control group. The increased urinary albumin was involved in the occurrence of epithelial casts and glomerulosclerosis in the kidney. We assessed vacuolization of kidney tissue and epithelial casts using H&E staining (Figure 1A) and glomerular hypertrophy and mesangial cells using PAS staining. The injuries were significantly increased in the control, ARA, and ARA+DHA groups, although it was decreased in the DHA group (Figure 1B). The results indicated that DHA protects kidney and attenuates the decreasing renal function.

### Fatty Acid Composition of Total Lipids in the Red Blood Cell Membrane, Plasma, and Kidneys

To assess the effects of PUFA-containing diet, we examined the FA composition of total lipids in red blood cell membrane, plasma, and kidneys of 5/6 nephrectomized rats at 4 weeks after nephrectomy.

The main FAs in the red blood cell membrane were C14:0, C16:0, C16:1, C18:0, C18:1, C18:2*ω*-6, C20:4*ω*-6, C20:5*ω*-3, C22:6*ω*-3, and C24:0 (Supplemental Table 3). The increased level of C20:4*ω*-6 or C22:6*ω*-3 in red blood cell membrane was due to the specific food diet. The C18:2*ω*-6 level was significantly decreased in the ARA and ARA+DHA groups compared with that in the control group. The C20:4*ω*-6 level was significantly increased in the ARA and ARA+DHA groups than in the control group. C22:6*ω*-3 levels in the ARA group were significantly decreased than in the control group, whereas they were significantly increased in the DHA and ARA+DHA groups compared with that in the control group.

The main FAs in plasma were C14:0, C16:0, C16:1, C18:0, C18:1, C18:2*ω*-6, C18:3*ω*-3, C20:4*ω*-6, C20:5*ω*-3, and C22:6*ω*-3 (Supplemental Table 4). The C18:2*ω*-6 level was significantly decreased in the ARA and ARA+DHA groups than in the control group. The C20:4*ω*-6 level was significantly increased in the ARA and ARA+DHA groups than in the control group. The C22:6*ω*-3 level was significantly increased in the DHA and ARA+DHA groups than in the control group.

Supplemental Table 5 presents the FA composition of total lipids in the kidney at 4 weeks after nephrectomy. The main FAs in the kidney were C14:0, C16:0, C16:1, C18:0, C18:1, C18:2*ω*-6, C20:4*ω*-6, C20:5*ω*-3, C22:6*ω*-3, and C24:0. The C18:2*ω*-6 level was significantly decreased in the ARA and ARA+DHA groups than in the control group. The C20:4*ω*-6 level was significantly decreased in the control and DHA groups than in the CS group. The C22:6*ω*-3 level was significantly increased in the DHA and ARA+DHA groups than in the CS and control groups. The results collectively indicated that feeding on a diet containing ARA and/or DHA could change the FA composition and increase the specific PUFA concentration in red blood cell membrane and kidney.

### DHA Attenuated the Increased Oxidative Stress and Uremic Toxin Levels in 5/6 Nephrectomized Rats at 4 Weeks after Nephrectomy

**Figure 2:**
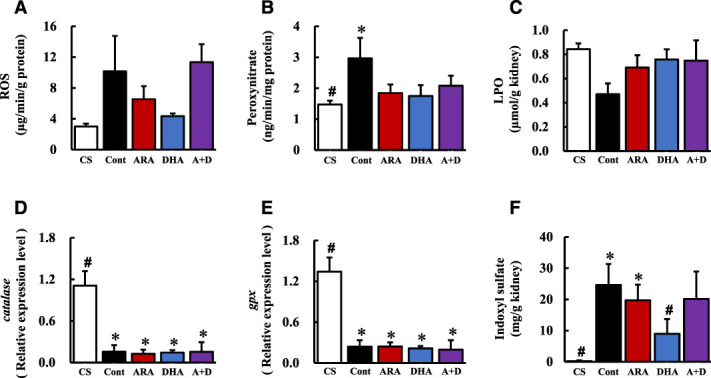
**DHA attenuated the increased oxidative stress and uremic toxin levels in 5/6 nephrectomized rats at 4 weeks after nephrectomy.** DHA attenuated the increased oxidative stress in kidney at 4 weeks after nephrectomy. (A) ROS, (B) ONOO^−^, (C) LPO, (D) catalase mRNA expression, (E) gpx mRNA expression, and (F) IS levels in kidney. Values are presented as the mean±SEM (*n*=5–7). *P < 0.05, by ANOVA and Tukey HSD (versus CS group). #*P* < 0.05, by ANOVA and Tukey HSD (versus control group). DHA, docosahexaenoic acid; ROS, reactive oxygen species; LPO, lipid peroxide; gpx, glutathione peroxidase; IS, indoxyl sulfate; CS, control sham; ARA, arachidonic acid.

ROS and ONOO^−^ levels in the kidney were increased in the control group than in the CS group, and they were attenuated in the DHA group than in the control group (Figure 2, A and B). Supplemental Table 6 presents the significant correlations between renal function and oxidative stress. A significant positive correlation was found between urinary albumin and ROS (*r*=0.4019, *P* = 0.0340), and significant negative correlations were found between Ccr and ROS (*r*=−0.4633, *P* = 0.0130) Ccr and ONOO^−^ (*r*=−0.4440, *P* = 0.0179). LPO in the kidney was decreased by nephrectomy, although it was not significantly different across the four groups of 5/6 nephrectomy rats (Figure 2C). The results indicated that ROS and ONOO^−^, implicated in oxidative stress in the kidney, decreased renal function at 4 weeks after nephrectomy.

To assess the cause of oxidative stress in the kidney at 4 weeks after nephrectomy, we investigated the mRNA expression of antioxidant enzymes, such as catalase and glutathione peroxidase, and the amount of IS in kidney. Catalase and glutathione peroxidase mRNA expression in kidney were significantly decreased due to nephrectomy, although it was not significantly different across the four groups of 5/6 nephrectomized rats (Figure 2, D and E). IS is known to cause ROS overproduction and is increased in the serum of patients with chronic renal failure.^20^ We determined the IS levels in the kidney using LC/MS/MS; the levels were found to be significantly increased in the control group than in the CS group at 4 weeks after nephrectomy (Figure 2F). Supplemental Table 6 presents the significant correlations between IS and oxidative stress in the kidney. Significant positive correlations were found between IS and ROS in the kidney (*r*=0.4472, *P* = 0.0170). The results indicated that ROS increase involves IS accumulation in the kidney at 4 weeks after nephrectomy. A DHA diet could attenuate IS accumulation, although ARA-containing and ARA+DHA-containing diets had no effect.

### DHA Attenuated Kidney Fibrosis at 4 Weeks after Nephrectomy

**Figure 3:**
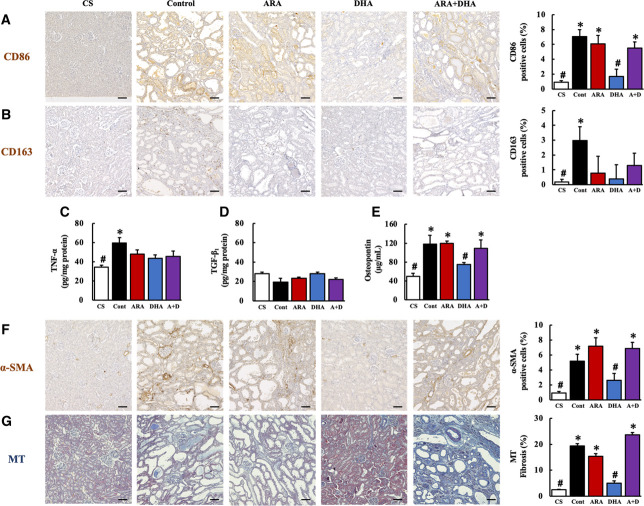
**DHA attenuated kidney fibrosis at 4 weeks after nephrectomy.** DHA attenuated inflammation in kidney and kidney fibrosis at 4 weeks after nephrectomy. (A) CD86, (B) CD163, (C) TNF-*α*, (D) TGF-*β*_1_, (E) urinary osteopontin, (F) *α*-SMA expression in kidney, and (G) kidney fibrosis using MT staining. Scale bar, 50 *µ*m. Values are presented as the mean±SEM (*n*=5–7). *P < 0.05, by ANOVA and Tukey HSD (versus CS group). #*P* < 0.05, by ANOVA and Tukey HSD (versus control group). DHA, docosahexaenoic acid; MT, Masson trichrome; CS, control sham; ARA, arachidonic acid.

IS has been reported to activate macrophages that are involved in kidney fibrosis and glomerulosclerosis. CD86 (M1 macrophage)–positive and CD163 (M2 macrophage)–positive cells in the kidney were assessed using immunohistochemical analysis (Figure 3, A and B). The expression of CD86 (7.03% in control group >0.94% in CS group) and CD163 (2.97% in control group >0.19% in CS group) was significantly increased in the control group than in the CS group at 4 weeks after nephrectomy. CD86 expression was decreased on feeding on a DHA diet, whereas CD163 showed no change due to feeding on any PUFA. The results indicated significantly increased CD86 expression over CD163 expression, and the kidneys of 5/6 nephrectomized rats were probably affected in the proinflammatory phase at 4 weeks after nephrectomy.

We further assessed TNF-*α* and urinary osteopontin levels, as proinflammatory cytokines, and TGF-*β*_1_ levels, as a resolution or fibrotic cytokine. TNF-*α* and urinary osteopontin levels were significantly increased in the control group than in the CS group at 4 weeks after nephrectomy and were decreased in the DHA group than in the control group (Figure 3, C and E); however, TGF-*β*_1_ was not significantly different across the five groups (Figure 3D). Increased TNF-*α* levels in the kidney have been reported to be involved in increased *α*-SMA expression and kidney fibrosis. We examined *α*-SMA expression and kidney fibrosis by MT staining of the kidney coronal sections. Both *α*-SMA expression and kidney fibrosis were significantly increased in the control group, ARA group, and ARA+DHA group than in the CS group; however, they were significantly decreased in the DHA group (Figure 3, F and G). Supplemental Table 6 presents the significant positive correlations found between TNF-*α* and ROS (*r*=0.5892, *P* = 0.0010), TNF-*α* and ONOO^−^ (*r*=0.5847, *P* = 0.0001), TNF-*α* and IS (*r*=0.6073, *P* = 0.0006), and TNF-*α* and urinary albumin (*r*=0.5334, *P* = 0.0035). These results suggested that proteinuria occurs with IS accumulation, followed by increase in oxidative stress, and promotion of inflammation and fibrosis at 4 weeks after nephrectomy. DHA-containing diet might attenuate such kidney injury.

### Fatty Acid Metabolite Levels in the Kidney

We examined the concentrations of PUFA metabolites in the kidney of 5/6 nephrectomized rats at 4 weeks after nephrectomy using LC/MS/MS. ARA metabolites are known to be involved in the development and maintenance of normal renal functions, and DHA metabolites play anti-inflammatory roles and protect the kidney. ARA, DHA, and EPA metabolite levels were found to be significantly decreased due to nephrectomy (Tables 3–5), although ARA metabolites were increased in the ARA group and DHA and EPA metabolites were increased in the DHA group compared with those in the control group. The results indicated that PUFA diet supplies each fatty acid metabolite to the kidney of 5/6 nephrectomized rats. Supplemental Table 7 presents the significant correlations between fatty acid metabolites and the parameters of renal function, oxidative stress, inflammation, and kidney fibrosis. Among the ARA metabolites, TXB2 was significant positive correlations with the parameters of renal function, oxidative stress, inflammation, and kidney fibrosis. On the other hand, DHA and EPA metabolites were significant negative correlation with these parameters.

**Table 3:** All arachidonic acid metabolite levels (mg/g kidney) in the kidney

Category	Metabolite	Sham	Nephrectomy			
		Control	Control	ARA	DHA	ARA+DHA
ARA	tetranor-PGFM	0.00±0.00	0.01±0.01	0.01±0.01	0.00±0.00	0.00±0.00
ARA	tetranor-PGEM	0.04±0.01	0.32±0.12	0.37±0.15	0.18±0.10	0.10±0.04
ARA	tetranor-PGDM	0.01±0.01	0.01±0.01	0.03±0.01	0.01±0.01	0.01±0.01
ARA	tetranor-PGJM	0.00±0.00	0.00±0.00	0.00±0.00	0.00±0.00	0.00±0.00
ARA	tetranor-PGAM	0.05±0.01	0.08±0.02	0.11±0.03	0.06±0.01	0.06±0.01
ARA	20-hydroxy-PGF2*α* or 19-hydroxy-PGF2*α*	0.01±0.01	0.01±0.00	0.00±0.00	0.01±0.01	0.00±0.00
ARA	20-hydroxy-PGE2	0.00±0.00	0.00±0.00	0.00±0.00	0.00±0.00	0.00±0.00
ARA	18-carboxy-dinor-LTB4	0.00±0.00	0.00±0.00	0.00±0.00	0.00±0.00	0.00±0.00
ARA	13,14-dihydro-15-keto-tetranor-PGF1*β*	0.00±0.00	0.00±0.00	0.00±0.00	0.00±0.00	0.01±0.01
ARA	2,3-dinor-8-iso-PGF2*α*	0.00±0.00	0.00±0.00	0.01±0.01	0.00±0.00	0.00±0.00
ARA	2,3-dinor-TXB2	0.00±0.00	0.00±0.00	0.00±0.00	0.00±0.00	0.00±0.00
ARA	13,14-dihydro-15-keto-tetranor-PGF1*α*	0.01±0.01	0.00±0.00	0.00±0.00	0.00±0.00	0.00±0.00
ARA	2,3-dinor-11b-PGF2*α*	0.00±0.00	0.00±0.00	0.00±0.00	0.00±0.00	0.00±0.00
ARA	6-keto-PGF1*α*	0.45±0.12^*^	2.58±0.40^*^	2.18±0.52^*^	0.65±0.19^*^	1.30±0.24^*^
ARA	13,14-dihydro-15-keto-tetranor-PGD2	0.00±0.00	0.00±0.00	0.00±0.00	0.00±0.00	0.00±0.00
ARA	20-carboxy-LTB4	0.00±0.00	0.00±0.00	0.00±0.00	0.00±0.00	0.00±0.00
ARA	20-hydroxy-LTB4	0.00±0.00	0.00±0.00	0.00±0.00	0.00±0.00	0.00±0.00
ARA	11-dehydro-2,3-dinor-TXB2	0.00±0.00	0.00±0.00	0.00±0.00	0.00±0.00	0.00±0.00
ARA	13,14-dihydro-15-keto-tetranor-PGE2	0.00±0.00	0.00±0.00	0.00±0.00	0.00±0.00	0.00±0.00
ARA	6,15-diketo-13,14-dihydro-PGF1*α*	0.05±0.02	0.04±0.01	0.02±0.01	0.02±0.01	0.03±0.02
ARA	iPF2*α*-IV	0.00±0.00	0.00±0.00	0.00±0.00	0.00±0.00	0.00±0.00
ARA	8-iso-15(R)-PGF2*α*	0.07±0.01^*^	0.02±0.01^*^	0.02±0.01^*^	0.02±0.01^*^	0.02±0.01^*^
ARA	8-iso-PGF2*α*	0.13±0.01^*^	0.04±0.01^*^	0.06±0.01^*^	0.05±0.01^*^	0.05±0.02^*^
ARA	TXB2	1.28±0.25^*^	3.71±0.73^*^	3.17±0.80^*^	0.81±0.19^*^	2.28±0.51^*^
ARA	11*β*-PGF2*α*	0.08±0.01^*^	0.04±0.01^*^	0.04±0.00^*^	0.03±0.00^*^	0.03±0.02^*^
ARA	5-iPF2*α*-VI	0.18±0.04^*^	0.01±0.01^*^	0.05±0.03^*^	0.11±0.04^*^	0.06±0.02^*^
ARA	8-iso-15-keto-PGF2*α*	0.08±0.02^*^	0.04±0.02^*^	0.00±0.00^*^	0.02±0.01^*^	0.03±0.02^*^
ARA	PGF2*α*	0.50±0.10^*^	0.18±0.05^*^	0.29±0.09^*^	0.15±0.01^*^	0.27±0.10^*^
ARA	8-iso-13,14-dihydro-15-keto-PGF2*α*	0.00±0.00	0.00±0.00	0.00±0.00	0.00±0.00	0.00±0.00
ARA	8-iso-PGE2	0.56±0.37	0.04±0.02	0.05±0.03	0.08±0.04	0.38±0.35
ARA	PGE2	2.21±0.63	1.23±0.43	2.58±0.72	0.83±0.13	1.56±0.26
ARA	11-dehydro-TXB2	0.00±0.00	0.00±0.00	0.00±0.00	0.00±0.00	0.01±0.01
ARA	15-keto-PGF2*α*	0.03±0.01	0.02±0.01	0.01±0.01	0.01±0.01	0.02±0.02
ARA	11*β*-PGE2	0.73±0.14	0.58±0.08	0.78±0.21	0.31±0.05	0.51±0.10
ARA	5S,14R-LXB4	0.00±0.00	0.00±0.00	0.00±0.00	0.00±0.00	0.01±0.01
ARA	PGK 2.00	0.00±0.00	0.00±0.00	0.00±0.00	0.00±0.00	0.00±0.00
ARA	PGD2	1.21±0.20	1.08±0.29	1.37±0.20	0.59±0.20	1.09±0.43
ARA	15-keto-PGF1*α*	0.00±0.00	0.00±0.00	0.00±0.00	0.00±0.00	0.00±0.00
ARA	11*β*-13,14-dihydro-15-keto-PGF2*α*	0.00±0.00	0.00±0.00	0.00±0.00	0.00±0.00	0.00±0.00
ARA	15-keto-PGE2	0.05±0.03	0.01±0.01	0.00±0.00	0.00±0.00	0.03±0.03
ARA	13,14-dihydro-PGF1*α*	0.00±0.00	0.00±0.00	0.00±0.00	0.00±0.00	0.00±0.00
ARA	14,15-LTC4	0.00±0.00	0.01±0.01	0.00±0.00	0.00±0.00	0.00±0.00
ARA	13,14-dihydro-15-keto-PGF2*α*	0.21±0.07^*^	0.05±0.03^*^	0.05±0.01^*^	0.04±0.00^*^	0.05±0.02^*^
ARA	5S,6R-LXA4	0.18±0.06	0.04±0.02	0.04±0.01	0.14±0.08	0.09±0.07
ARA	13,14-dihydro-15-keto-PGE2	0.39±0.10	0.10±0.03	0.13±0.05	0.09±0.03	0.07±0.04
ARA	5S,6S-LXA4	0.11±0.05	0.01±0.01	0.04±0.01	0.05±0.02	0.04±0.04
ARA	14,15-LTE4	0.00±0.00	0.00±0.00	0.00±0.00	0.00±0.00	0.00±0.00
ARA	13,14-dihydro-15-keto-PGD2	0.09±0.03	0.02±0.01	0.04±0.01	0.03±0.00	0.04±0.03
ARA	LTC4	0.00±0.00	0.00±0.00	0.00±0.00	0.00±0.00	0.00±0.00
ARA	11-trans-LTC4	0.00±0.00	0.00±0.00	0.00±0.00	0.00±0.00	0.00±0.00
ARA	LTD4	0.00±0.00	0.00±0.00	0.00±0.00	0.00±0.00	0.00±0.00
ARA	LTE4	0.00±0.00	0.00±0.00	0.00±0.00	0.00±0.00	0.00±0.00
ARA	LTF4	0.00±0.00	0.00±0.00	0.00±0.00	0.00±0.00	0.00±0.00
ARA	8-iso-PGA2	0.01±0.01	0.02±0.01	0.01±0.01	0.01±0.01	0.00±0.00
ARA	11-trans-LTD4	0.00±0.00	0.00±0.00	0.00±0.00	0.00±0.00	0.00±0.00
ARA	PGA2	0.00±0.00	0.00±0.00	0.00±0.00	0.01±0.01	0.01±0.01
ARA	PGJ2	0.01±0.01	0.01±0.01	0.01±0.01	0.02±0.01	0.01±0.01
ARA	11-trans-LTE4	0.00±0.00	0.00±0.00	0.00±0.00	0.00±0.00	0.00±0.00
ARA	PGB2	0.00±0.00	0.00±0.00	0.00±0.00	0.00±0.00	0.00±0.00
ARA	8,12-iso-iPF2*α*-VI-1,5-lactone	0.04±0.01^*^	0.00±0.00^*^	0.02±0.01^*^	0.11±0.03^*^	0.04±0.02^*^
ARA	8,15-DiHETE	0.00±0.00	0.00±0.00	0.00±0.00	0.00±0.00	0.00±0.00
ARA	6-trans-LTB4	0.03±0.01	0.00±0.00	0.01±0.01	0.02±0.02	0.02±0.01
ARA	5,15-DiHETE	0.25±0.03^*^	0.06±0.03^*^	0.11±0.01^*^	0.16±0.06^*^	0.10±0.05^*^
ARA	13,14-dihydro-15-keto-PGA2	0.02±0.01	0.00±0.00	0.00±0.00	0.01±0.01	0.01±0.01
ARA	LTB4	0.03±0.01	0.01±0.01	0.01±0.00	0.03±0.02	0.01±0.01
ARA	13,14-dihydro-15-keto PGJ2	0.03±0.02	0.01±0.01	0.02±0.01	0.03±0.02	0.02±0.01
ARA	12-keto-LTB4	0.00±0.00	0.00±0.00	0.00±0.00	0.02±0.01	0.01±0.01
ARA	tetranor-12-HETE	0.00±0.00	0.00±0.00	0.00±0.00	0.00±0.00	0.00±0.00
ARA	*N*-acetyl-LTE4	0.00±0.00	0.00±0.00	0.00±0.00	0.00±0.00	0.00±0.00
ARA	LTB3	0.00±0.00	0.00±0.00	0.00±0.00	0.00±0.00	0.00±0.00
ARA	14,15-DHET	3.44±0.37	2.08±0.20	4.88±1.00	3.68±1.24	4.96±1.81
ARA	12-HHT	0.55±0.11^*^	0.16±0.02^*^	0.21±0.11^*^	0.29±0.04^*^	0.41±0.15^*^
ARA	11,12-DHET	4.62±0.66^*^	1.30±0.23^*^	3.31±0.63^*^	2.74±0.87^*^	3.30±0.99^*^
ARA	8,9-DHET	1.00±0.16	0.33±0.06	0.75±0.16	0.66±0.22	0.86±0.27
ARA	20-carboxy-ARA	0.02±0.01	0.00±0.00	0.00±0.00	0.00±0.00	0.00±0.00
ARA	5,6-DHET	0.22±0.03^*^	0.09±0.01^*^	0.18±0.05^*^	0.17±0.05^*^	0.27±0.01^*^
ARA	19-HETE	0.05±0.04	0.00±0.00	0.02±0.02	0.00±0.00	0.04±0.02
ARA	15-deoxy-delta-12,14-PGJ2	0.01±0.01	0.04±0.01	0.04±0.02	0.06±0.06	0.03±0.01
ARA	20-HETE	0.00±0.00	0.00±0.00	0.00±0.00	0.00±0.00	0.00±0.00
ARA	18-HETE	0.00±0.00	0.00±0.00	0.00±0.00	0.00±0.00	0.01±0.01
ARA	17-HETE	0.01±0.01	0.00±0.00	0.01±0.01	0.01±0.01	0.00±0.00
ARA	16-HETE	0.16±0.02^*^	0.06±0.02^*^	0.07±0.02^*^	0.09±0.01^*^	0.08±0.04^*^
ARA	15-HETE	7.31±0.64^*^	3.01±0.71^*^	3.75±0.42^*^	3.52±0.54^*^	3.40±1.22^*^
ARA	11-HETE	7.50±0.73^*^	6.45±0.44^*^	5.84±0.84^*^	3.62±0.47^*^	4.85±1.16^*^
ARA	8-HETE	0.83±0.10^*^	0.27±0.08^*^	0.34±0.05^*^	0.40±0.06^*^	0.40±0.15^*^
ARA	15-KETE	1.24±0.48	0.30±0.17	0.20±0.03	0.36±0.18	0.52±0.35
ARA	15-HpETE	0.45±0.29	0.10±0.10	0.01±0.01	0.05±0.04	0.12±0.11
ARA	12-HETE	3.81±0.50^*^	1.73±0.31^*^	2.39±0.43^*^	2.04±0.26^*^	2.51±0.78^*^
ARA	9-HETE	0.32±0.03^*^	0.11±0.03^*^	0.14±0.04^*^	0.19±0.03^*^	0.15±0.07^*^
ARA	5-HETE	6.27±0.42^*^	2.20±0.66^*^	2.87±0.47^*^	3.80±1.07^*^	2.50±0.95^*^
ARA	12-HpETE	0.09±0.05	0.04±0.04	0.00±0.00	0.01±0.01	0.03±0.02
ARA	12-KETE	0.17±0.10	0.04±0.03	0.01±0.01	0.01±0.01	0.05±0.04
ARA	5,6-DHET-lactone	0.31±0.02^*^	0.35±0.04^*^	0.50±0.09^*^	0.43±0.12^*^	0.68±0.09^*^
ARA	5-HpETE	0.02±0.01	0.01±0.01	0.00±0.00	0.00±0.00	0.01±0.01
ARA	14,15-EET	0.70±0.10	0.65±0.08	0.97±0.18	0.71±0.22	0.68±0.24
ARA	5-KETE	0.80±0.20	0.23±0.10	0.22±0.05	0.38±0.20	0.37±0.24
ARA	11,12-EET	0.23±0.03	0.22±0.02	0.34±0.06	0.23±0.07	0.29±0.09
ARA	8,9-EET	0.23±0.02	0.22±0.02	0.34±0.06	0.23±0.07	0.25±0.07
ARA	5,6-EET	0.31±0.05	0.23±0.03	0.37±0.07	0.25±0.07	0.36±0.09
ARA	ARA	9.89±0.59^*^	8.22±0.21^*^	7.44±0.33^*^	5.78±0.83^*^	5.67±0.92^*^

Values are presented as the mean±SEM (*n*=5–7). ARA, arachidonic acid; DHA, docosahexaenoic acid; TXB2, Thromboxane B2; EET, epoxyeicosatrienoic acids.

* Significant differences were observed between the experimental group and the CS group, as determined by ANOVA and Tukey HSD tests (*P* < 0.01).

**Table 4:** All docosahexaenoic acid metabolite levels (mg/g kidney) in the kidney

Category	Metabolite	Sham	Nephrectomy			
		Control	Control	ARA	DHA	ARA+DHA
DHA	Resolvin D3	0.00±0.00	0.00±0.00	0.00±0.00	0.00±0.00	0.00±0.00
DHA	Resolvin D2	0.00±0.00	0.00±0.00	0.00±0.00	0.00±0.00	0.00±0.00
DHA	Resolvin D1	0.00±0.00	0.00±0.00	0.00±0.00	0.00±0.00	0.00±0.00
DHA	Resolvin D4	0.00±0.00	0.00±0.00	0.00±0.00	0.00±0.00	0.00±0.00
DHA	Maresin1	0.00±0.00	0.00±0.00	0.00±0.00	0.00±0.00	0.00±0.00
DHA	10,17-DiHDHA	0.01±0.00	0.01±0.01	0.00±0.00	0.02±0.01	0.01±0.01
DHA	Resolvin D5	0.02±0.01	0.01±0.01	0.00±0.00	0.01±0.01	0.02±0.01
DHA	7,17-hydroxy-DPA	0.00±0.00	0.00±0.00	0.00±0.00	0.00±0.00	0.00±0.00
DHA	19,20-DiHDPA	0.24±0.02^*^	0.08±0.01^*^	0.12±0.02^*^	0.32±0.11^*^	0.26±0.08^*^
DHA	20-HDHA	0.42±0.03^*^	0.15±0.04^*^	0.16±0.02^*^	0.49±0.08^*^	0.28±0.12^*^
DHA	16-HDHA	0.70±0.05^*^	0.32±0.08^*^	0.24±0.03	0.79±0.08^*^	0.50±0.20^*^
DHA	17-HDHA	0.13±0.02^*^	0.08±0.01^*^	0.06±0.01^*^	0.18±0.04^*^	0.17±0.05^*^
DHA	13-HDHA	0.52±0.04	0.42±0.06	0.23±0.04	0.53±0.08	0.55±0.15
DHA	10-HDHA	0.21±0.02^*^	0.08±0.02^*^	0.06±0.02	0.25±0.03^*^	0.19±0.07^*^
DHA	14-HDHA	0.32±0.06	0.18±0.03	0.12±0.02	0.38±0.07	0.39±0.16
DHA	11-HDHA	0.26±0.02^*^	0.11±0.03^*^	0.08±0.02	0.31±0.04^*^	0.23±0.09^*^
DHA	7-HDHA	0.06±0.01^*^	0.02±0.01^*^	0.01±0.01^*^	0.07±0.01^*^	0.03±0.02^*^
DHA	8-HDHA	0.13±0.02^*^	0.06±0.02^*^	0.05±0.01^*^	0.16±0.03^*^	0.13±0.03^*^
DHA	17-HpDHA	0.00±0.00	0.00±0.00	0.00±0.00	0.00±0.00	0.00±0.00
DHA	4-HDHA	0.93±0.05^*^	0.28±0.08^*^	0.25±0.02^*^	0.83±0.12^*^	0.61±0.23^*^
DHA	19,20-EpDPA	0.68±0.10^*^	0.77±0.11^*^	0.69±0.13^*^	1.69±0.47^*^	1.17±0.32^*^
DHA	16,17-EpDPA	0.17±0.03	0.23±0.04	0.15±0.03	0.42±0.14	0.27±0.08
DHA	DHA	12.7±1.11^*^	13.5±0.95^*^	7.29±0.62^*^	17.1±2.18^*^	11.9±1.56^*^

Values are presented as the mean±SEM (*n*=5–7). ARA, arachidonic acid; DHA, docosahexaenoic acid.

* Significant differences were observed between the experimental group and the CS group, as determined by ANOVA and Tukey HSD tests (*P* < 0.01).

**Table 5:** All eicosapentaenoic acid metabolite levels (mg/g kidney) in the kidney

Category	Metabolite	Sham	Nephrectomy			
		Control	Control	ARA	DHA	ARA+DHA
EPA	delta17-6-keto-PGF1*α*	0.01±0.01^*^	0.12±0.03^*^	0.04±0.01^*^	0.04±0.02^*^	0.04±0.01^*^
EPA	Resolvin E1	0.00±0.00	0.00±0.00	0.00±0.00	0.00±0.00	0.00±0.00
EPA	8-iso-PGF3*α*	0.03±0.01	0.04±0.01	0.02±0.01	0.05±0.01	0.02±0.01
EPA	TXB3	0.01±0.01^*^	0.08±0.03^*^	0.03±0.01^*^	0.01±0.01^*^	0.04±0.01^*^
EPA	PGF3*α*	0.00±0.00	0.00±0.00	0.00±0.00	0.00±0.00	0.00±0.00
EPA	11-dehydro-TXB3	0.00±0.00	0.00±0.00	0.00±0.00	0.00±0.00	0.00±0.00
EPA	PGE3	0.08±0.02	0.09±0.02	0.07±0.02	0.07±0.02	0.07±0.02
EPA	PGD3	0.16±0.03^*^	0.12±0.04^*^	0.08±0.02^*^	0.34±0.10^*^	0.16±0.08^*^
EPA	LXA5	0.00±0.00	0.00±0.00	0.00±0.00	0.01±0.01	0.00±0.00
EPA	LTB5	0.00±0.00	0.00±0.00	0.00±0.00	0.00±0.00	0.00±0.00
EPA	17,18-DiHETE	0.49±0.05^*^	0.25±0.04^*^	0.33±0.06^*^	1.07±0.31^*^	0.50±0.16^*^
EPA	14,15-DiHETE	0.28±0.03^*^	0.22±0.05^*^	0.36±0.09^*^	1.25±0.44^*^	0.57±0.29^*^
EPA	5,6-DiHETE	0.03±0.02	0.00±0.00	0.01±0.01	0.05±0.03	0.03±0.01
EPA	18-HEPE	0.47±0.07^*^	0.22±0.08^*^	0.16±0.03^*^	0.70±0.15^*^	0.23±0.11^*^
EPA	15-HEPE	0.11±0.03	0.07±0.02	0.05±0.02	0.18±0.03	0.11±0.04
EPA	11-HEPE	0.08±0.01	0.09±0.02	0.05±0.02	0.11±0.01	0.08±0.02
EPA	8-HEPE	0.01±0.00	0.00±0.00	0.00±0.00	0.00±0.00	0.00±0.00
EPA	9-HEPE	0.00±0.00	0.00±0.00	0.00±0.00	0.00±0.00	0.00±0.00
EPA	12-HEPE	0.33±0.06^*^	0.20±0.05^*^	0.19±0.05^*^	0.60±0.07^*^	0.36±0.13^*^
EPA	5-HEPE	0.42±0.04^*^	0.21±0.07^*^	0.19±0.05^*^	0.92±0.16^*^	0.25±0.10^*^
EPA	15-HpEPE	0.00±0.00	0.00±0.00	0.00±0.00	0.00±0.00	0.00±0.00
EPA	12-HpEPE	0.00±0.00	0.00±0.00	0.00±0.00	0.00±0.00	0.00±0.00
EPA	5-HpEPE	0.00±0.00	0.00±0.00	0.00±0.00	0.00±0.00	0.00±0.00
EPA	17,18-EpETE	0.15±0.04^*^	0.26±0.05^*^	0.29±0.06^*^	0.66±0.21^*^	0.23±0.13^*^
EPA	14,15-EpETE	0.16±0.03^*^	0.21±0.03^*^	0.23±0.05^*^	0.66±0.20^*^	0.25±0.12^*^
EPA	EPA	2.36±0.22^*^	2.76±0.15^*^	1.63±0.19^*^	4.43±0.56^*^	1.74±0.37^*^

Values are presented as the mean±SEM (*n*=5–7). ARA, arachidonic acid; DHA, docosahexaenoic acid; EPA, eicosapentaenoic acid; HEPE, hydroxy eicosapentaenoic acid.

* Significant differences were observed between the experimental group and the CS group, as determined by ANOVA and Tukey HSD tests (*P* < 0.01).

## Discussion

Our previous study had reported that dietary ARA or DHA attenuates the increased oxidative stress in plasma 4 weeks after nephrectomy and contributes to suppress the progression of renal failure at 16 weeks after nephrectomy.^17^ Therefore, in this study, we focused on the effects of oxidative stress and renal function in the kidney 4 weeks after nephrectomy. Our previous study had reported that the increased urinary albumin excretion in 5/6 nephrectomized rats was attenuated on feeding on ARA-containing and DHA-containing diets.^17^ However, in this study, we found urinary albumin excretion to be attenuated by DHA-containing diet, not by ARA-containing diet, at 4 weeks after nephrectomy (Table 1). Therefore, we suggested that the effect of attenuation of decreasing renal function varies between ARA and DHA. Urinary albumin excretion causes glomerular diseases, such as podocyte injury, glomerulosclerosis by mesangial hypertrophy, dysfunction of vascular endothelial cells, formation of coagulate precipitation, and epithelial casts in tubule.^21^ With H&E, PAS, and MT staining, we found coagulate precipitation, glomerular hypertrophy, and fibrosis in the kidney coronal section to have significantly increased at 4 weeks after nephrectomy (Figure 1, A and B, and Figure 3G); however, kidney injury was attenuated by feeding on a DHA-containing diet. In this study, we did not observe the podocytes or assess the function of vascular endothelial cells; these will need to be investigated in future.

ROS levels in the kidney were found to be significantly increased at 4 weeks after nephrectomy (Figure 2A). The result was in line with our previous study (increase of LPO level in plasma at 4 weeks after nephrectomy^17^). Moreover, we found ROS levels in the kidney to be associated with decreased renal function. Oxidative stress occurs with overproduction of ROS and/or reduction in antioxidant defense capacity.^15,16^ Therefore, we assessed the mRNA expression of antioxidant enzyme and IS levels that cause ROS overproduction in the kidney at 4 weeks after nephrectomy and found the mRNA expression of an antioxidant enzyme to be decreased by nephrectomy, although PUFA diet had no affect (Figure 2, D and E). IS was increased by nephrectomy but was attenuated by DHA-containing diet (Figure 2F). IS has been reported to be excreted from kidney under normal renal function conditions; however, IS accumulates in the kidney when renal function is decreased, and CKD progresses because of the activation of NADPH oxidase and overproduction of ROS in mesangial cells and renal proximal tubular cells.^22^ We found that a DHA-containing diet could attenuate IS accumulation in the kidney after nephrectomy. IS has been reported as a protein-bound uremic toxin whose dialytic clearance is low.^23^ *In vitro* dialysis model demonstrated that DHA increased the removal rate of IS from IS-accumulated cell. Therefore, in this study, we suggested that feeding DHA to the rats could suppress ROS overproduction by increased IS removal rate and attenuate IS accumulation in the kidney. Increasing ROS induction by IS led to the occurrence of inflammation and kidney fibrosis.^22^ Activated M1 macrophage is reported to increase TNF-*α*, while activated M2 macrophage increases TGF-*β*_1_. These cause an increase in *α*-SMA levels, leading to kidney fibrosis.^24^ In this study, TNF-*α* and *α*-SMA levels in the kidney were increased by nephrectomy. Moreover, we found that parameters, such as IS, ROS, TNF-*α*, and *α*-SMA, in the kidney were significantly correlated. However, TGF-*β*_1_ in the kidney was not increased by nephrectomy. Therefore, we suggested that TNF-*α* increase by IS induced ROS overproduction in the kidney, which is involved in kidney fibrosis. On the other hand, feeding DHA-containing diet decreased the parameters that had been increased by nephrectomy. Therefore, we found that intake of DHA-containing diet can suppress IS and ROS induced TNF-*α* increasing and kidney fibrosis.

The effects of ARA+DHA diet were not consistent with our previous study. In our previous study, ARA, DHA, and ARA+DHA diets were found to suppress the decreased renal function. However, in this study, ARA-containing diets had no suppressive effect after nephrectomy. As the reason, we consider that influence of different kind of PUFA metabolite in the kidney. *ω*-6 and *ω*-3 fatty acid metabolites are known to be associated with proinflammatory phase and resolution phase, respectively.^25^ TXA2 has been reported to be a precursor metabolite of TXB2 that is produced in the proinflammatory phase. It has involved in inflammatory eicosanoids and leads to platelet aggregation and glomerular hypertrophy by glomerular hyperfiltration.^26^ TXB2 is overproduced by oxidative stress and has inflammatory effects, such as vasoconstriction and vascular endothelial cells injury.^27^ We found that the TXB2 level was increased after nephrectomy and ARA diet to be involved in oxidative stress, inflammation, and fibrosis at 4 weeks after nephrectomy. Therefore, in this study, we suggested that ARA-containing diet did not suppress renal function reduction at 4 weeks after nephrectomy. On the other hand, we found that DHA metabolites, such as 20-HDHA and 4-HDHA, and EPA metabolites, such as resolvin E and 18-HEPE, increased in the kidney because of dietary DHA. A DHA metabolite, namely protectin D, is an anti-inflammatory factor 2 and protects against kidney diseases 4–14. The EPA metabolite, 18-HEPE, preserves cardiac function under pressure overload. Our results indicated that DHA and EPA metabolites are involved in the suppression of oxidative stress, inflammation, and fibrosis at 4 weeks after nephrectomy. This is the first study that reported the eicosanoid and docosanoid levels in the kidney of 5/6 nephrectomized rats fed dietary ARA and DHA. We found that DHA metabolites are involved in the attenuation of oxidative stress, inflammation, and fibrosis in the kidney. However, we were not assessed which DHA metabolites can suppress oxidative stress, inflammation, and kidney fibrosis in this study. In the future, we need to comprehensively assess that *in vitro* of IS-induced CKD model used to suppress effects on IS accumulation, oxidative stress, and kidney fibrosis by DHA metabolites.

In conclusion, the results of this study indicated that DHA may have suppressive effects on the progression of renal failure. One possible mechanism would be *via* the suppression of IS accumulation, oxidative stress, and kidney fibrosis. Therefore, diets containing DHA might suppress IS accumulation, oxidative stress, and fibrosis in the kidney at 4 weeks after nephrectomy.

## Supplementary Material

SUPPLEMENTARY MATERIAL

## Data Availability

All data is included in the manuscript and/or supporting information.

## Supplemental Material

Supplemental Table 1. LC/MS/MS setting for indoxyl sulfate.

Supplemental Table 2. List of primer sequences.

Supplemental Table 3. Fatty acid composition of red blood cell membrane at 4 weeks after nephrectomy.

Supplemental Table 4. Fatty acid composition of plasma at 4 weeks after nephrectomy.

Supplemental Table 5. Fatty acid composition of total lipid in kidney at 4 weeks after nephrectomy.

Supplemental Table 6. Correlation of all of parameters at 4 weeks after nephrectomy.

Supplemental Table 7. Correlation of all of arachidonic acid metabolites and all of parameters at 4 weeks after nephrectomy.

Supplemental Table 8. Correlation of all of docosahexaenoic acid metabolites and all of parameters at 4 weeks after nephrectomy.

Supplemental Table 9. Correlation of all of eicosapentaenoic acid metabolites and all of parameters at 4 weeks after nephrectomy.
