# Supplementary material for: Suppressing Effects of Docosahexaenoic Acid–Containing Diets on Oxidative Stress and Fibrosis in 5/6 Nephrectomized Rats
Source: Kidney360. 2023 May 24;4(12):1690–701. doi: 10.34067/KID.0000000000000152 (PMC10758513; doi:10.34067/KID.0000000000000152)
Supplement: SUPPLEMENTARY MATERIAL [file kidney360-4-1690-s001.pdf]

Table S1. LC/MS/MS setting for IS

uremic toxin	ion mode	Q1 (*m/z*)	Q3 (*m/z*)	Q1 Pre Vias (V)	CE (V)	Q3 Pre Vias (V)
IS	negative	212.1	132.0	22.0	22.0	13.0

Table S2. List of primers sequence

	Forward	Reverse
catalase	5'-TGCTGCCTTTTCTGTTCCTT-3'	5'-AAGGTGCTGGGTAGGGAAGT-3'
glutathione peroxidase	5'-TTGACCCTAACCAAGGATGC-3'	5'-CACCCCTTCTGCGTTGTATT-3'
18S	5'-AAACGGCTACCACATCCAAG-3'	5'-CCTCCAATGGATCCTCGTTA-3'

Table S3. Fatty acid composition of red blood cell membrane at 4 weeks after nephrectomy

(%)	Sham	Nephrectomy			
	control	control	ARA	DHA	ARA+DHA
C14:0	0.3 ± 0.1	0.4 ± 0.1	0.3 ± 0.2	0.3 ± 0.1	0.3 ± 0.1
C16:0	33.9 ± 0.9	32.3 ± 1.7	32.5 ± 1.5	33.2 ± 0.6	33.6 ± 1.4
C16:1	0.6 ± 0.1 ^{a}	0.7 ± 0.2 ^{a}	0.3 ± 0.1 ^{b}	0.3 ± 0.1 ^{b}	0.4 ± 0.1 ^{b}
C18:0	16.8 ± 0.7 ^{a,b}	17.0 ± 1.4 ^{b}	18.0 ± 1.0 ^{a}	16.2 ± 0.8 ^{b}	15.9 ± 0.6 ^{b}
C18:1	8.3 ± 0.4 ^{a}	10.3 ± 2.6 ^{b}	8.3 ± 0.5 ^{a,b}	7.8 ± 0.2 ^{a}	7.6 ± 0.7 ^{a}
C18:2ω-6	6.0 ± 0.5 ^{a}	6.9 ± 1.4 ^{a}	4.1 ± 0.3 ^{b}	6.8 ± 0.5 ^{a}	3.3 ± 0.3 ^{b}
C20:4ω-6	26.0 ± 0.6 ^{b,c}	24.0 ± 1.6 ^{c,d}	30.3 ± 1.0 ^{a}	23.2 ± 1.2 ^{d}	27.4 ± 0.9 ^{b}
C20:5ω-3	1.1 ± 0.3 ^{a,b}	1.2 ± 0.5 ^{a,b}	0.4 ± 0.1 ^{c}	1.5 ± 0.4 ^{a}	0.7 ± 0.5 ^{b,c}
C22:6ω-3	4.7 ± 0.8 ^{a}	4.9 ± 0.5 ^{a}	3.8 ± 0.3 ^{a}	7.7 ± 0.4 ^{b}	7.0 ± 0.3 ^{b}
C24:0	1.5 ± 0.1 ^{c}	1.7 ± 0.5 ^{a,b,c}	1.5 ± 0.2 ^{b,c}	2.0 ± 0.4 ^{a,b}	2.1 ± 0.3 ^{a}
ω-6/ ω-3	5.6 ± 0.4	5.1 ± 0.2	8.1 ± 0.4	3.7 ± 0.1	4.0 ± 0.1

C14:0, myristic acid; C16:0, palmitic acid; C16:1, palmitoleic acid; C18:0, stearic acid; C18:1, oleic acid; C18:2ω-6, linoleic acid; C20:4ω-6, arachidonic acid; C20:5ω-3, eicosapentaenoic acid; C22:6ω-3, docosahexaenoic acid; C24:0, lignoceric acid; ω-6/ω-3, ratio of ω-6 and ω-3. Values are presented as the mean ± SEM (n = 5-7). a-d, by ANOVA and Tukey HSD test ($P < 0.01$).

Table S4. Fatty acid composition of plasma at 4 weeks after nephrectomy

(%)	Sham	Nephrectomy			
	control	control	ARA	DHA	ARA+DHA
C14:0	0.8 ± 0.1	0.7 ± 0.1	0.6 ± 0.1	0.9 ± 0.1	0.6 ± 0.1
C16:0	28.2 ± 0.9 [a,b]	25.0 ± 1.3 [a,b]	24.1 ± 0.8 [b]	28.7 ± 1.0 [a]	27.1 ± 1.1 [a,b]
C16:1	3.5 ± 0.2	2.5 ± 0.3	2.2 ± 0.4	3.5 ± 0.6	2.6 ± 0.4
C18:0	10.5 ± 0.6	11.5 ± 1.0	11.8 ± 0.6	10.3 ± 0.5	11.6 ± 0.9
C18:1	19.0 ± 1.2	18.1 ± 1.1	16.4 ± 0.8	19.2 ± 1.0	5.9 ± 1.2
C18:2ω-6	14.1 ± 0.4 [b]	17.1 ± 0.7 [a]	10.5 ± 0.5 [c]	16.7 ± 0.6 [a]	9.8 ± 0.7 [c]
C18:3ω-3	1.8 ± 0.2 [a]	1.7 ± 0.5 [a,b]	2.0 ± 0.2 [a]	1.1 ± 0.2 [a,b]	0.7 ± 0.2 [b]
C20:4ω-6	15.6 ± 1.4 [a]	16.2 ± 1.5 [a]	28.2 ± 1.0 [b]	11.3 ± 1.1 [a]	24.4 ± 2.2 [b]
C20:5ω-3	1.5 ± 0.2 [a,b]	2.7 ± 0.6 [a]	0.8 ± 0.4 [b]	2.2 ± 0.2 [a,b]	1.3 ± 0.4 [a,b]
C22:6ω-3	1.3 ± 0.2 [a]	1.8 ± 0.3 [a]	1.3 ± 0.2 [a]	3.5 ± 0.3 [a,b]	4.3 ± 1.2 [b]
ω-6/ ω-3	4.9 ± 0.6 [a]	5.1 ± 0.4 [a]	10.5 ± 1.4 [b]	3.1 ± 0.1 [a]	4.9 ± 0.7 [a]

C14:0, myristic acid; C16:0, palmitic acid; C16:1, palmitoleic acid; C18:0, stearic acid; C18:1, oleic acid; C18:2ω-6, linoleic acid; C18:3ω-3, α-linolenic acid; C20:4ω-6, arachidonic acid; C20:5ω-3, eicosapentaenoic acid; C22:6ω-3, docosahexaenoic acid; ω-6/ω-3, ratio of ω-6 and ω-3. Values are presented as the mean ± SEM (n = 5-7). a-d, by ANOVA and Tukey HSD test ($P < 0.01$).

Table S5. Fatty acid composition of total lipid in kidney at 4 weeks after nephrectomy

(%)	Sham	Nephrectomy			
	control	control	ARA	DHA	ARA+DHA
C14:0	0.6 ± 0.1	0.9 ± 0.1	0.7 ± 0.1	0.6 ± 0.2	0.8 ± 0.2
C16:0	24.4 ± 1.1	26.4 ± 1.4	24.8 ± 1.0	26.4 ± 1.0	26.8 ± 1.0
C16:1	1.7 ± 0.3	2.5 ± 0.4	1.7 ± 0.3	2.1 ± 0.4	2.2 ± 0.4
C18:0	16.2 ± 0.8	13.3 ± 1.5	16.1 ± 1.2	14.2 ± 1.1	15.2 ± 1.1
C18:1	11.9 ± 1.5	19.9 ± 2.6	15.9 ± 2.3	16.9 ± 2.4	15.8 ± 2.4
C18:2ω-6	11.2 ± 0.3 [a]	11.8 ± 0.8 [a]	8.8 ± 0.5 [b]	13.4 ± 0.5 [a]	8.0 ± 0.5 [b]
C20:4ω-6	25.1 ± 1.9 [a]	15.8 ± 2.5 [b]	22.7 ± 2.4 [a,b]	15.0 ± 2.4 [b]	21.3 ± 2.4 [a,b]
C20:5ω-3	0.9 ± 0.1 [a]	1.2 ± 0.3 [a]	0.9 ± 0.1 [a]	2.4 ± 0.1 [b]	1.0 ± 0.1 [a]
C22:6ω-3	1.9 ± 0.1 [a]	1.6 ± 0.2 [a]	1.7 ± 0.2 [b]	3.1 ± 0.2 [b]	2.8 ± 0.2 [b]
C24:0	1.8 ± 0.3 [a]	1.4 ± 0.2 [a,b]	1.7 ± 0.2 [a]	1.6 ± 0.2 [a,b]	1.6 ± 0.2 [a,b]
ω-6/ ω-3	13.4 ± 0.6	11.0 ± 1.1	12.7 ± 0.8	5.3 ± 0.3	7.6 ± 0.2

C14:0, myristic acid; C16:0, palmitic acid; C16:1, palmitoleic acid; C18:0, stearic acid; C18:1, oleic acid; C18:2ω-6, linoleic acid; C20:4ω-6, arachidonic acid; C20:5ω-3, eicosapentaenoic acid; C22:6ω-3, docosahexaenoic acid; C24:0, lignoceric acid; ω-6/ω-3. ratio of ω-6 and ω-3. Values are presented as the mean ± SEM (n = 5-7). a-b, by ANOVA and Tukey HSD test ($P < 0.01$).

Table S6. Correlation of all of parameters at 4 weeks after nephrectomy

Parameter	C_{cr}		U-ALB		ROS		ONOO-		IS		TNF-α		TGF-$β_1$		MT		α-SMA		CD86		CD163	
	r	*P*	*r*	*P*	*r*	*P*	*r*	*P*	*r*	*P*	*r*	*P*	*r*	*P*	*r*	*P*	*r*	*P*	*r*	*P*	*r*	*P*
C_{cr}			-0.569	0.002 *	-0.463	0.013 *	-0.444	0.018 *	-0.633	0.000 *	-0.661	0.000 *	0.482	0.010 *	-0.741	<.0001 *	-0.752	<.0001 *	-0.742	<.0001 *	-0.515	0.017 *
U-ALB	-0.569	0.002 *			0.402	0.034 *	0.143	0.467	0.905	<.0001 *	0.533	0.004 *	-0.533	0.004 *	0.364	0.062 *	0.443	0.021 *	0.648	0.001 *	0.010	0.966
ROS	-0.463	0.013 *	0.402	0.034 *			0.218	0.266	0.447	0.017 *	0.589	0.001 *	-0.723	<.0001 *	0.684	<.0001 *	0.551	0.003 *	0.446	0.038 *	0.568	0.007 *
ONOO-	-0.444	0.018 *	0.143	0.467	0.218	0.266			0.273	0.161	0.585	0.001 *	-0.111	0.576	0.489	0.010 *	0.308	0.118	0.649	0.001 *	0.866	<.0001 *
IS	-0.633	0.000 *	0.905	<.0001 *	0.447	0.017 *	0.273	0.161			0.607	0.001 *	-0.624	0.000 *	0.497	0.008 *	0.436	0.023 *	0.659	0.001 *	0.160	0.490
TNF-α	-0.661	0.000 *	0.533	0.004 *	0.589	0.001 *	0.585	0.001 *	0.607	0.001 *			-0.417	0.027 *	0.581	0.002 *	0.561	0.002 *	0.788	<.0001 *	0.854	<.0001 *
TGF-$β_1$	0.482	0.010 *	-0.533	0.004 *	-0.723	<.0001 *	-0.111	0.576	-0.624	0.000 *	-0.417	0.027 *			-0.585	0.001 *	-0.368	0.059	-0.549	0.008 *	-0.033	0.887
MT	-0.741	<.0001 *	0.364	0.062	0.684	<.0001 *	0.489	0.010 *	0.497	0.008 *	0.581	0.002 *	-0.585	0.001 *			0.761	<.0001 *	0.709	0.000 *	0.641	0.002 *
α-SMA	-0.752	<.0001 *	0.443	0.021 *	0.551	0.003 *	0.308	0.118	0.436	0.023 *	0.561	0.002 *	-0.368	0.059	0.761	<.0001 *			0.730	0.000 *	0.499	0.021 *
CD86	-0.742	<.0001 *	0.648	0.001 *	0.446	0.038 *	0.649	0.001 *	0.659	0.001 *	0.788	<.0001 *	-0.549	0.008 *	0.709	0.000 *	0.730	0.000 *			0.741	0.000 *
CD163	-0.515	0.017 *	0.010	0.966	0.568	0.007 *	0.866	<.0001 *	0.160	0.490	0.854	<.0001 *	-0.033	0.887	0.641	0.002 *	0.499	0.021 *	0.741	0.000 *		

Correlations between parameters ($P < 0.05$). *P* value by Pearson's correlation coefficient.

Table S7. Correlation between all of ARA metabolites and all of parameters at 4 weeks after nephrectomy

Category	Metabolites	Renal function						Oxidative stress									Inflammation and fibrosis																			
		Ccr			Urinary albumin			ROS			ONOO-			IS			TNF-α			TGF-β_1			MT			α-SMA			CD86			CD163		
		r	*P*		*r*	*P*		*r*	*P*		*r*	*P*		*r*	*P*		*r*	*P*		*r*	*P*		*r*	*P*		*r*	*P*		*r*	*P*		*r*	*P*	
ARA	tetranor-PGFM	-0.1962	0.3170		0.2190	0.2629		0.4966	0.0072	*	-0.1569	0.4252		0.2093	0.2851		0.2549	0.1905		-0.4797	0.0098	*	0.1988	0.3201		0.4375	0.0225	*	0.4336	0.0438	*	-0.1336	0.5638	
ARA	tetranor-PGEM	-0.4278	0.0231	*	0.2241	0.2517		0.4599	0.0138	*	0.2350	0.2287		0.4922	0.0078	*	0.4603	0.0137	*	-0.5148	0.0051	*	0.4185	0.0298	*	0.3340	0.0886		0.3953	0.0686		0.1920	0.4044	
ARA	tetranor-PGDM	-0.0079	0.9683		0.1259	0.5233		0.0020	0.9919		-0.0878	0.6567		0.0186	0.9250		0.0179	0.9281		0.1946	0.3211		-0.0422	0.8343		0.2580	0.1939		0.1453	0.5188		-0.1212	0.6008	
ARA	tetranor-PGJM	0.0000	1.0000		0.0000	1.0000		0.0000	1.0000		0.0000	1.0000		0.0000	1.0000		0.0000	1.0000		0.0000	1.0000		0.0000	1.0000		0.0000	1.0000		0.0000	1.0000		0.0000	1.0000	
ARA	tetranor-PGAM	-0.4275	0.0233	*	0.4217	0.0254	*	0.4096	0.0304	*	0.1199	0.5435		0.5083	0.0057	*	0.4901	0.0081	*	-0.4187	0.0266	*	0.3157	0.1086		0.4129	0.0323	*	0.5109	0.0151	*	0.1991	0.3868	
ARA	20-hydroxy-PGF2α or 19-hydroxy-PGF2α	0.1630	0.4072		-0.1872	0.3401		-0.2099	0.2837		0.0285	0.8854		-0.2433	0.2122		-0.1072	0.5871		0.1862	0.3427		-0.1486	0.4594		-0.1482	0.4606		0.0667	0.7681		-0.0231	0.9207	
ARA	20-hydroxy-PGE2	0.0000	1.0000		0.0000	1.0000		0.0000	1.0000		0.0000	1.0000		0.0000	1.0000		0.0000	1.0000		0.0000	1.0000		0.0000	1.0000		0.0000	1.0000		0.0000	1.0000		0.0000	1.0000	
ARA	18-carboxy-dinor-LTB4	0.0000	1.0000		0.0000	1.0000		0.0000	1.0000		0.0000	1.0000		0.0000	1.0000		0.0000	1.0000		0.0000	1.0000		0.0000	1.0000		0.0000	1.0000		0.0000	1.0000		0.0000	1.0000	
ARA	13,14-dihydro-15-keto-tetranor-PGF1β	-0.1397	0.4782		0.0327	0.8689		0.4106	0.0300	*	0.1875	0.3393		0.0364	0.8543		0.1063	0.5904		-0.1454	0.4605		0.3216	0.1019		0.2358	0.2363		0.0000	1.0000		0.0000	1.0000	
ARA	2,3-dinor-8-iso-PGF2α	0.0796	0.6872		0.0071	0.9714		0.0168	0.9323		-0.0398	0.8405		-0.0986	0.6176		0.0162	0.9349		0.0929	0.6383		-0.1220	0.5443		-0.0317	0.8753		-0.0025	0.9913		-0.1276	0.5814	
ARA	2,3-dinor-TXB2	0.0000	1.0000		0.0000	1.0000		0.0000	1.0000		0.0000	1.0000		0.0000	1.0000		0.0000	1.0000		0.0000	1.0000		0.0000	1.0000		0.0000	1.0000		0.0000	1.0000		0.0000	1.0000	
ARA	13,14-dihydro-15-keto-tetranor-PGF1α	0.3774	0.0477	*	-0.1750	0.3731		-0.0692	0.7264		-0.1686	0.3912		-0.1806	0.3576		-0.0861	0.6631		0.3020	0.1183		-0.2192	0.2719		-0.2200	0.2702		-0.2343	0.2939		-0.0872	0.7070	
ARA	2,3-dinor-11b-PGF2α	0.0000	1.0000		0.0000	1.0000		0.0000	1.0000		0.0000	1.0000		0.0000	1.0000		0.0000	1.0000		0.0000	1.0000		0.0000	1.0000		0.0000	1.0000		0.0000	1.0000		0.0000	1.0000	
ARA	6-keto-PGF1α	-0.6224	0.0004	*	0.5318	0.0036	*	0.3359	0.0805		0.4616	0.0134	*	0.6266	0.0004	*	0.6817	<.0001	*	-0.5687	0.0016	*	0.5930	0.0011	*	0.4172	0.0304	*	0.7204	0.0002	*	0.5040	0.0198	*
ARA	13,14-dihydro-15-keto-tetranor-PGD2	0.0000	1.0000		0.0000	1.0000		0.0000	1.0000		0.0000	1.0000		0.0000	1.0000		0.0000	1.0000		0.0000	1.0000		0.0000	1.0000		0.0000	1.0000		0.0000	1.0000		0.0000	1.0000	
ARA	20-carboxy-LTB4	0.0000	1.0000		0.0000	1.0000		0.0000	1.0000		0.0000	1.0000		0.0000	1.0000		0.0000	1.0000		0.0000	1.0000		0.0000	1.0000		0.0000	1.0000		0.0000	1.0000		0.0000	1.0000	
ARA	20-hydroxy-LTB4	0.0000	1.0000		0.0000	1.0000		0.0000	1.0000		0.0000	1.0000		0.0000	1.0000		0.0000	1.0000		0.0000	1.0000		0.0000	1.0000		0.0000	1.0000		0.0000	1.0000		0.0000	1.0000	
ARA	11-dehydro-2,3-dinor-TXB2	0.0000	1.0000		0.0000	1.0000		0.0000	1.0000		0.0000	1.0000		0.0000	1.0000		0.0000	1.0000		0.0000	1.0000		0.0000	1.0000		0.0000	1.0000		0.0000	1.0000		0.0000	1.0000	
ARA	13,14-dihydro-15-keto-tetranor-PGE2	0.0000	1.0000		0.0000	1.0000		0.0000	1.0000		0.0000	1.0000		0.0000	1.0000		0.0000	1.0000		0.0000	1.0000		0.0000	1.0000		0.0000	1.0000		0.0000	1.0000		0.0000	1.0000	
ARA	6,15-diketo-13,14-dihydro-PGF1α	0.3421	0.0748		-0.1104	0.5761		-0.0013	0.9949		-0.1982	0.3119		-0.1969	0.3152		-0.1750	0.3730		0.1117	0.5713		-0.1682	0.4016		-0.2384	0.2310		-0.2830	0.2018		-0.1698	0.4617	
ARA	iPF2α-IV	0.0000	1.0000		0.0000	1.0000		0.0000	1.0000		0.0000	1.0000		0.0000	1.0000		0.0000	1.0000		0.0000	1.0000		0.0000	1.0000		0.0000	1.0000		0.0000	1.0000		0.0000	1.0000	
ARA	8-iso-15(R)-PGF2α	0.7436	<.0001	*	-0.4816	0.0095	*	-0.2526	0.1946		-0.2923	0.1312		-0.5275	0.0039	*	-0.4691	0.0118	*	0.3907	0.0398	*	-0.4594	0.0159	*	-0.4543	0.0173	*	-0.5500	0.0080	*	-0.3778	0.0914	
ARA	8-iso-PGF2α	0.7108	<.0001	*	-0.3979	0.0360	*	-0.2635	0.1756		-0.3389	0.0777		-0.4233	0.0248	*	-0.5290	0.0038	*	0.1785	0.3636		-0.4821	0.0109	*	-0.5344	0.0041	*	-0.6201	0.0021	*	-0.5182	0.0161	*
ARA	TXB2	-0.5170	0.0048	*	0.2717	0.1619		0.4666	0.0123	*	0.6896	<.0001	*	0.4064	0.0319	*	0.6837	<.0001	*	-0.4373	0.0200	*	0.6639	0.0002	*	0.4810	0.0111	*	0.6191	0.0021	*	0.7783	<.0001	*
ARA	11b-PGF2α	0.5787	0.0013	*	-0.3175	0.0996		-0.1174	0.5519		-0.3123	0.1057		-0.3249	0.0916		-0.3878	0.0414	*	0.1511	0.4427		-0.3903	0.0442	*	-0.4756	0.0122	*	-0.4380	0.0415	*	-0.4648	0.0338	*
ARA	5-iPF2α-VI	0.5832	0.0011	*	-0.4995	0.0068	*	-0.2894	0.1352		-0.3140	0.1037		-0.5265	0.0040	*	-0.6035	0.0007	*	0.2153	0.2713		-0.5300	0.0045	*	-0.5363	0.0039	*	-0.6360	0.0015	*	-0.4298	0.0518	
ARA	8-iso-15-keto-PGF2α	0.5352	0.0033	*	-0.3690	0.0533		-0.1733	0.3779		-0.1383	0.4827		-0.4284	0.0229	*	-0.4027	0.0336	*	0.2403	0.2181		-0.3391	0.0836		-0.4755	0.0122	*	-0.5787	0.0048	*	-0.2467	0.2811	
ARA	PGF2α	0.4631	0.0131	*	-0.4243	0.0244	*	-0.0115	0.9536		-0.1038	0.5991		-0.3147	0.1028		-0.3030	0.1170		0.0289	0.8840		-0.1164	0.5633		-0.2326	0.2430		-0.4125	0.0564		-0.0565	0.8079	
ARA	8-iso-13,14-dihydro-15-keto-PGF2α	0.0000	1.0000		0.0000	1.0000		0.0000	1.0000		0.0000	1.0000		0.0000	1.0000		0.0000	1.0000		0.0000	1.0000		0.0000	1.0000		0.0000	1.0000		0.0000	1.0000		0.0000	1.0000	
ARA	8-iso-PGE2	0.3385	0.0780		-0.1851	0.3457		0.1160	0.5568		-0.0872	0.6591		-0.2056	0.2940		-0.0676	0.7326		0.2465	0.2061		-0.0855	0.6716		-0.1227	0.5421		-0.3108	0.1592		-0.1178	0.6110	
ARA	PGE2	0.1082	0.5835		-0.2681	0.1677		-0.1686	0.3910		0.0921	0.6412		-0.1107	0.5748		-0.2006	0.3061		0.0429	0.8283		0.0475	0.8139		-0.0327	0.8715		-0.1061	0.6385		0.2174	0.3437	

Correlations between ARA metabolites and parameters (P < 0.05). P value by Pearson's correlation coefficient.

Table S7. Correlation between all of ARA metabolites and all of parameters at 4 weeks after nephrectomy

Category	Metabolites	Renal function				Oxidative stress						Inflammation and fibrosis																			
		Ccr		Urinary albumin		ROS		ONOO-		IS		TNF-α		TGF-β_1		MT		α-SMA		CD86		CD163	
		r	*P*	*r*	*P*	*r*	*P*	*r*	*P*	*r*	*P*	*r*	*P*	*r*	*P*	*r*	*P*	*r*	*P*	*r*	*P*	*r*	*P*
ARA	11-dehydro-TXB2	0.0966	0.6247	-0.0803	0.6845	0.2750	0.1567	0.0768	0.6976	-0.0827	0.6757	0.0207	0.9169	0.0113	0.9544	0.1212	0.5469	0.0487	0.8092	-0.2520	0.2578	-0.1427	0.5372
ARA	15-keto-PGF2α	0.2051	0.2951	-0.2720	0.1614	0.0321	0.8710	0.1145	0.5617	-0.2890	0.1357	-0.0982	0.6190	0.0916	0.6429	0.0022	0.9912	-0.0826	0.6820	-0.2600	0.2425	0.3060	0.1774
ARA	11b-PGE2	0.1340	0.4965	-0.1842	0.3480	0.1080	0.5842	0.1112	0.5731	-0.0034	0.9863	-0.0318	0.8725	-0.2930	0.1303	0.1414	0.4817	-0.0602	0.7656	-0.0554	0.8064	0.0278	0.9047
ARA	5S,14R-LXB4	-0.1397	0.4782	0.0327	0.8689	0.4106	0.0300 *	0.1875	0.3393	0.0364	0.8543	0.1063	0.5904	-0.1454	0.4605	0.3216	0.1019	0.2358	0.2363	0.0000	1.0000	0.0000	1.0000
ARA	PGK 2.00	0.0000	1.0000	0.0000	1.0000	0.0000	1.0000	0.0000	1.0000	0.0000	1.0000	0.0000	1.0000	0.0000	1.0000	0.0000	1.0000	0.0000	1.0000	0.0000	1.0000	0.0000	1.0000
ARA	PGD2	0.0573	0.7720	-0.0686	0.7287	0.3039	0.1159	-0.0931	0.6375	-0.0132	0.9470	0.0593	0.7645	-0.1956	0.3185	0.1914	0.3388	0.1290	0.5212	0.0139	0.9511	0.0177	0.9393
ARA	15-keto-PGF1α	0.3445	0.0726	-0.1751	0.3729	-0.0973	0.6223	-0.1243	0.5286	-0.1838	0.3491	-0.1079	0.5847	0.2123	0.2780	-0.2305	0.2474	-0.2347	0.2386	-0.2520	0.2578	-0.1427	0.5372
ARA	11b-13,14-dihydro-15-keto-PGF2α	-0.0165	0.9335	-0.0725	0.7138	-0.0558	0.7781	0.0027	0.9889	-0.1000	0.6125	-0.0831	0.6743	-0.0459	0.8164	-0.0052	0.9795	-0.1314	0.5135	0.0953	0.6732	-0.0509	0.8267
ARA	15-keto-PGE2	0.2983	0.1232	-0.1840	0.3487	0.1054	0.5935	-0.0603	0.7604	-0.2109	0.2814	-0.1505	0.4446	0.1263	0.5218	-0.0513	0.7994	-0.1069	0.5956	-0.2632	0.2367	-0.1078	0.6417
ARA	13,14-dihydro-PGF1α	0.0000	1.0000	0.0000	1.0000	0.0000	1.0000	0.0000	1.0000	0.0000	1.0000	0.0000	1.0000	0.0000	1.0000	0.0000	1.0000	0.0000	1.0000	0.0000	1.0000	0.0000	1.0000
ARA	14,15-LTC4	-0.1277	0.5173	0.1798	0.3598	-0.1134	0.5655	0.2060	0.2930	0.0952	0.6299	0.1098	0.5780	-0.0020	0.9920	0.0271	0.8933	-0.0801	0.6914	0.2757	0.2143	-0.0722	0.7557
ARA	13,14-dihydro-15-keto-PGF2α	0.6169	0.0005 *	-0.3309	0.0854	-0.2489	0.2014	-0.2561	0.1883	-0.3703	0.0524	-0.4062	0.0320 *	0.2886	0.1364	-0.3924	0.0429 *	-0.4167	0.0306 *	-0.4283	0.0467 *	-0.2814	0.2166
ARA	5S,6R-LXA4	0.3776	0.0476 *	-0.3307	0.0856	-0.0382	0.8472	-0.2766	0.1541	-0.3409	0.0758	-0.2778	0.1524	0.1683	0.3920	-0.3232	0.1001	-0.3864	0.0465 *	-0.4847	0.0222 *	-0.2767	0.2246
ARA	13,14-dihydro-15-keto-PGE2	0.6484	0.0002 *	-0.4055	0.0323 *	-0.2490	0.2013	-0.2239	0.2521	-0.4200	0.0261 *	-0.4530	0.0155 *	0.2064	0.2921	-0.4506	0.0183 *	-0.4926	0.0090 *	-0.5061	0.0163 *	-0.2691	0.2382
ARA	5S,6S-LXA4	0.4340	0.0210 *	-0.3190	0.0980	-0.0259	0.8960	-0.2211	0.2581	-0.3277	0.0887	-0.1973	0.3143	0.2918	0.1318	-0.2758	0.1638	-0.2920	0.1394	-0.4537	0.0339 *	-0.2373	0.3004
ARA	14,15-LTE4	-0.0402	0.8392	-0.1175	0.5515	0.0371	0.8514	0.0143	0.9424	-0.1046	0.5962	-0.1950	0.3200	-0.0947	0.6318	0.2600	0.1903	0.1057	0.5999	-0.0339	0.8810	-0.0451	0.8462
ARA	13,14-dihydro-15-keto-PGD2	0.5384	0.0031 *	-0.3384	0.0781	-0.0849	0.6677	-0.2643	0.1741	-0.3659	0.0555	-0.2958	0.1265	0.2536	0.1929	-0.3099	0.1157	-0.3027	0.1249	-0.5374	0.0099 *	-0.3444	0.1264
ARA	LTC4	0.0000	1.0000	0.0000	1.0000	0.0000	1.0000	0.0000	1.0000	0.0000	1.0000	0.0000	1.0000	0.0000	1.0000	0.0000	1.0000	0.0000	1.0000	0.0000	1.0000	0.0000	1.0000
ARA	11-trans-LTC4	0.0000	1.0000	0.0000	1.0000	0.0000	1.0000	0.0000	1.0000	0.0000	1.0000	0.0000	1.0000	0.0000	1.0000	0.0000	1.0000	0.0000	1.0000	0.0000	1.0000	0.0000	1.0000
ARA	LTD4	0.0000	1.0000	0.0000	1.0000	0.0000	1.0000	0.0000	1.0000	0.0000	1.0000	0.0000	1.0000	0.0000	1.0000	0.0000	1.0000	0.0000	1.0000	0.0000	1.0000	0.0000	1.0000
ARA	LTE4	0.0000	1.0000	0.0000	1.0000	0.0000	1.0000	0.0000	1.0000	0.0000	1.0000	0.0000	1.0000	0.0000	1.0000	0.0000	1.0000	0.0000	1.0000	0.0000	1.0000	0.0000	1.0000
ARA	LTF4	0.0000	1.0000	0.0000	1.0000	0.0000	1.0000	0.0000	1.0000	0.0000	1.0000	0.0000	1.0000	0.0000	1.0000	0.0000	1.0000	0.0000	1.0000	0.0000	1.0000	0.0000	1.0000
ARA	8-iso-PGA2	0.1278	0.5168	-0.1083	0.5834	-0.1235	0.5313	-0.0777	0.6944	-0.1138	0.5643	-0.2716	0.1620	0.0000	1.0000	-0.1774	0.3759	-0.3359	0.0867	-0.1847	0.4106	-0.3476	0.1226
ARA	11-trans-LTD4	0.0000	1.0000	0.0000	1.0000	0.0000	1.0000	0.0000	1.0000	0.0000	1.0000	0.0000	1.0000	-0.1859	0.3436	0.0000	1.0000	0.0000	1.0000	0.0000	1.0000	0.0000	1.0000
ARA	PGA2	-0.0727	0.7132	-0.1619	0.4105	-0.0774	0.6954	-0.2592	0.1829	-0.1808	0.3573	-0.0487	0.8056	0.0000	1.0000	-0.0503	0.8032	-0.1153	0.5670	-0.1114	0.6215	0.0521	0.8224
ARA	PGJ2	-0.0925	0.6398	-0.2614	0.1791	-0.2355	0.2276	0.0611	0.7576	-0.1610	0.4130	0.0829	0.6751	0.0963	0.6259	0.0032	0.9874	-0.0347	0.8635	0.1034	0.6469	0.4540	0.0387 *
ARA	11-trans-LTE4	0.0000	1.0000	0.0000	1.0000	0.0000	1.0000	0.0000	1.0000	0.0000	1.0000	0.0000	1.0000	0.1332	0.4992	0.0000	1.0000	0.0000	1.0000	0.0000	1.0000	0.0000	1.0000
ARA	PGB2	0.0000	1.0000	0.0000	1.0000	0.0000	1.0000	0.0000	1.0000	0.0000	1.0000	0.0000	1.0000	0.0000	1.0000	0.0000	1.0000	0.0000	1.0000	0.0000	1.0000	0.0000	1.0000
ARA	8,12-iso-iPF2a-VI-1,5-lactone	0.1781	0.3646	-0.2715	0.1622	-0.1371	0.4865	-0.2589	0.1835	-0.3473	0.0702	-0.2746	0.1573	0.0000	1.0000	-0.2641	0.1832	-0.2230	0.2635	-0.5129	0.0147 *	-0.3172	0.1611
ARA	8,15-DiHETE	0.0000	1.0000	0.0000	1.0000	0.0000	1.0000	0.0000	1.0000	0.0000	1.0000	0.0000	1.0000	0.4738	0.0109 *	0.0000	1.0000	0.0000	1.0000	0.0000	1.0000	0.0000	1.0000
ARA	6-trans-LTB4	0.3098	0.1086	-0.2276	0.2440	-0.1243	0.5285	-0.3470	0.0704	-0.2895	0.1351	-0.2967	0.1252	0.0000	1.0000	-0.2946	0.1358	-0.2886	0.1442	-0.3998	0.0652	-0.1685	0.4653

Correlations between ARA metabolites and parameters (P < 0.05). P value by Pearson's correlation coefficient.

Table S7. Correlation between all of ARA metabolites and all of parameters at 4 weeks after nephrectomy

Category	Metabolites	Renal function				Oxidative stress						Inflammation and fibrosis											
		Ccr		Urinary albumin		ROS		ONOO-		IS		TNF-α		TGF-β1		MT		α-SMA		CD86		CD163	
		r	*P*	*r*	*P*	*r*	*P*	*r*	*P*	*r*	*P*	*r*	*P*	*r*	*P*	*r*	*P*	*r*	*P*	*r*	*P*	*r*	*P*
ARA	5,15-DiHETE	0.6563	0.0001 *	-0.4949	0.0074 *	-0.2881	0.1371	-0.5030	0.0064 *	-0.5619	0.0019 *	-0.5761	0.0013 *	0.2164	0.2687	-0.5809	0.0015 *	-0.5551	0.0026 *	-0.7441	<.0001 *	-0.5486	0.0100 *
ARA	13,14-dihydro-15-keto-PGA2	0.4726	0.0111 *	-0.2910	0.1330	-0.0728	0.7127	-0.1521	0.4397	-0.3056	0.1138	-0.2396	0.2194	0.3519	0.0663	-0.3163	0.1080	-0.3529	0.0710	-0.4211	0.0510	-0.2120	0.3562
ARA	LTB4	0.3297	0.0866	-0.3093	0.1092	-0.1268	0.5201	-0.3281	0.0883	-0.3145	0.1031	-0.2970	0.1249	0.1666	0.3968	-0.3545	0.0696	-0.4040	0.0366 *	-0.4973	0.0185 *	-0.2597	0.2556
ARA	13,14-dihydro-15-keto PGJ2	0.2778	0.1523	-0.3021	0.1182	-0.0652	0.7418	-0.2270	0.2455	-0.2628	0.1767	-0.1496	0.4473	0.2092	0.2854	-0.2676	0.1772	-0.3355	0.0871	-0.3463	0.1144	-0.1623	0.4822
ARA	12-keto-LTB4	0.0794	0.6881	-0.1830	0.3513	0.0539	0.7855	-0.1304	0.5083	-0.2524	0.1950	-0.1013	0.6082	0.1349	0.4939	-0.1452	0.4699	-0.0728	0.7184	-0.4007	0.0646	-0.2126	0.3549
ARA	tetranor-12-HETE	0.0000	1.0000	0.0000	1.0000	0.0000	1.0000	0.0000	1.0000	0.0000	1.0000	0.0000	1.0000	0.3392	0.0774	0.0000	1.0000	0.0000	1.0000	0.0000	1.0000	0.0000	1.0000
ARA	N-acetyl-LTE4	0.0000	1.0000	0.0000	1.0000	0.0000	1.0000	0.0000	1.0000	0.0000	1.0000	0.0000	1.0000	0.0000	1.0000	0.0000	1.0000	0.0000	1.0000	0.0000	1.0000	0.0000	1.0000
ARA	LTB3	0.0000	1.0000	0.0000	1.0000	0.0000	1.0000	0.0000	1.0000	0.0000	1.0000	0.0000	1.0000	0.0000	1.0000	0.0000	1.0000	0.0000	1.0000	0.0000	1.0000	0.0000	1.0000
ARA	14,15-DHET	0.0413	0.8345	-0.1541	0.4337	-0.0627	0.7514	-0.2504	0.1988	-0.2947	0.1279	-0.3794	0.0465 *	0.0000	1.0000	0.0568	0.7784	0.0976	0.6280	-0.2214	0.3220	-0.2701	0.2363
ARA	12-HHT	0.4564	0.0146 *	-0.3713	0.0518	0.0121	0.9514	-0.1266	0.5208	-0.3521	0.0661	-0.4012	0.0343 *	0.1388	0.4812	-0.1549	0.4403	-0.3184	0.1056	-0.6152	0.0023 *	-0.3316	0.1420
ARA	11,12-DHET	0.5674	0.0016 *	-0.4279	0.0231 *	-0.2020	0.3026	-0.3992	0.0354 *	-0.5418	0.0029 *	-0.5802	0.0012 *	0.4408	0.0189 *	-0.3171	0.1070	-0.2913	0.1405	-0.5864	0.0041 *	-0.4671	0.0328 *
ARA	8,9-DHET	0.4737	0.0109 *	-0.3818	0.0450 *	-0.1652	0.4007	-0.3792	0.0466 *	-0.5013	0.0066 *	-0.5597	0.0020 *	0.4058	0.0321 *	-0.2272	0.2543	-0.2373	0.2334	-0.5466	0.0085 *	-0.4478	0.0418 *
ARA	20-carboxy-ARA	0.5663	0.0017 *	-0.2924	0.1311	-0.1921	0.3275	-0.2427	0.2134	-0.3091	0.1094	-0.2798	0.1493	0.2695	0.1655	-0.3673	0.0594	-0.3905	0.0440 *	-0.3892	0.0734	-0.1962	0.3941
ARA	5,6-DHET	0.2371	0.2245	-0.1840	0.3485	-0.0788	0.6902	-0.3207	0.0962	-0.3165	0.1008	-0.2236	0.2528	0.3911	0.0396 *	-0.0515	0.7987	0.0410	0.8390	-0.2736	0.2180	-0.0957	0.6797
ARA	19-HETE	0.3252	0.0913	-0.0992	0.6155	0.0705	0.7215	-0.1474	0.4540	-0.0776	0.6948	-0.1147	0.5611	0.2040	0.2977	-0.0917	0.6493	-0.1418	0.4804	-0.3000	0.1749	-0.1762	0.4450
ARA	15-deoxy-delta-12,14-PGJ2	-0.2669	0.1697	-0.0662	0.7378	-0.0135	0.9458	0.0440	0.8239	-0.0163	0.9345	0.0963	0.6259	-0.1635	0.4057	0.0300	0.8820	-0.0269	0.8940	0.1117	0.6206	0.1123	0.6280
ARA	20-HETE	0.2621	0.1778	-0.2343	0.2301	-0.1330	0.5000	-0.0862	0.6628	-0.2460	0.2071	-0.2076	0.2892	0.1403	0.4765	-0.2515	0.2056	-0.2153	0.2808	-0.2780	0.2103	-0.1821	0.4295
ARA	18-HETE	0.1176	0.5512	-0.0435	0.8262	-0.0581	0.7689	-0.1139	0.5638	-0.0872	0.6590	-0.3082	0.1106	-0.0475	0.8104	-0.0076	0.9700	-0.1427	0.4778	-0.1570	0.4854	-0.1382	0.5503
ARA	17-HETE	0.2813	0.1470	-0.1835	0.3500	-0.0835	0.6726	-0.1767	0.3683	-0.1645	0.4028	-0.0886	0.6540	0.2613	0.1792	-0.2456	0.2168	-0.1746	0.3837	-0.2559	0.2504	-0.1719	0.4563
ARA	16-HETE	0.6211	0.0004 *	-0.3419	0.0749	-0.2293	0.2405	-0.3806	0.0457 *	-0.4396	0.0192 *	-0.5128	0.0053 *	0.3485	0.0692	-0.4563	0.0167 *	-0.4899	0.0095 *	-0.7036	0.0003 *	-0.4958	0.0223 *
ARA	15-HETE	0.7427	<.0001 *	-0.5142	0.0051 *	-0.2381	0.2225	-0.3986	0.0356 *	-0.5607	0.0019 *	-0.5607	0.0019 *	0.2992	0.1220	-0.4913	0.0093 *	-0.5073	0.0069 *	-0.6856	0.0004 *	-0.4872	0.0251 *
ARA	11-HETE	0.3719	0.0514	-0.1391	0.4803	0.0827	0.6756	-0.0076	0.9692	-0.1238	0.5304	-0.1056	0.5928	-0.2037	0.2985	-0.0439	0.8277	-0.2435	0.2210	-0.1526	0.4979	-0.0954	0.6807
ARA	8-HETE	0.7322	<.0001 *	-0.4875	0.0085 *	-0.2004	0.3066	-0.4183	0.0267 *	-0.5389	0.0031 *	-0.5468	0.0026 *	0.3236	0.0930	-0.4763	0.0120 *	-0.5225	0.0052 *	-0.6965	0.0003 *	-0.5012	0.0206 *
ARA	15-KETE	0.4663	0.0124 *	-0.2977	0.1239	-0.0623	0.7527	-0.2137	0.2748	-0.3340	0.0823	-0.2737	0.1588	0.2395	0.2197	-0.2686	0.1756	-0.3047	0.1222	-0.3937	0.0698	-0.1851	0.4219
ARA	15-HpETE	0.3611	0.0591	-0.2235	0.2529	-0.0639	0.7465	-0.1743	0.3751	-0.2596	0.1822	-0.2268	0.2459	0.2007	0.3057	-0.1989	0.3199	-0.2224	0.2650	-0.2541	0.2537	-0.1019	0.6604
ARA	12-HETE	0.5597	0.0020 *	-0.2715	0.1623	-0.1059	0.5916	-0.3812	0.0454 *	-0.2556	0.1893	-0.4450	0.0176 *	0.1401	0.4771	-0.3146	0.1100	-0.3871	0.0461 *	-0.5924	0.0037 *	-0.4745	0.0298 *
ARA	9-HETE	0.7201	<.0001 *	-0.5247	0.0042 *	-0.1989	0.3103	-0.4671	0.0122 *	-0.5817	0.0012 *	-0.5458	0.0027 *	0.3475	0.0700	-0.4855	0.0103 *	-0.5198	0.0055 *	-0.7515	<.0001 *	-0.5253	0.0145 *
ARA	5-HETE	0.7267	<.0001 *	-0.5401	0.0030 *	-0.3012	0.1194	-0.5051	0.0061 *	-0.6072	0.0006 *	-0.6007	0.0007 *	0.2883	0.1367	-0.6149	0.0006 *	-0.6237	0.0005 *	-0.7329	0.0001 *	-0.5269	0.0141 *
ARA	12-HpETE	0.2906	0.1335	-0.2059	0.2933	-0.1188	0.5471	-0.1314	0.5051 *	-0.2660	0.1712	-0.2320	0.2349	0.1324	0.5020	-0.1740	0.3853	-0.1732	0.3876	-0.2159	0.3345	-0.0360	0.8768
ARA	12-KETE	0.3891	0.0407 *	-0.2119	0.2790	-0.0586	0.7672	-0.1774	0.3666	-0.2544	0.1915	-0.1896	0.3339	0.2385	0.2217	-0.1990	0.3196	-0.2224	0.2648	-0.2358	0.2907	-0.0967	0.6768
ARA	5,6-DHET-lactone	-0.2945	0.1282	0.2225	0.2552	0.0748	0.7052	-0.1302	0.5090	0.0610	0.7580	-0.1945	0.3212	0.0344	0.8620	0.3254	0.0977	0.3584	0.0664	0.0100	0.9647	-0.1132	0.6251
ARA	5-HpETE	0.2686	0.1669	-0.1733	0.3778	0.0279	0.8881	-0.0807	0.6831	-0.2143	0.2735	-0.1855	0.3446	0.1081	0.5839	-0.0743	0.7127	-0.1217	0.5455	-0.2343	0.2939	-0.0872	0.7070
ARA	14,15-EET	0.0435	0.8260	-0.0631	0.7498	-0.1655	0.4001	-0.1231	0.5327	-0.2231	0.2537	-0.2948	0.1278	0.1638	0.4050	-0.0353	0.8611	0.0597	0.7674	-0.1224	0.5873	-0.2532	0.2680
ARA	5-KETE	0.5083	0.0057 *	-0.3700	0.0526	-0.0829	0.6749	-0.3014	0.1190	-0.4156	0.0278 *	-0.3454	0.0719	0.2203	0.2600	-0.3517	0.0720	-0.3832	0.0485 *	-0.5104	0.0152 *	-0.3113	0.1695
ARA	11,12-EET	-0.0254	0.8977	0.0220	0.9116	-0.0565	0.7752	-0.1500	0.4460	-0.1108	0.5746	-0.2997	0.1213	0.0648	0.7430	0.0758	0.7070	0.1500	0.4552	-0.0584	0.7962	-0.3061	0.1772
ARA	8,9-EET	-0.0175	0.9297	-0.0624	0.7525	-0.1174	0.5518	-0.0844	0.6694	-0.2073	0.2898	-0.2784	0.1514	0.1372	0.4863	0.0410	0.8393	0.1297	0.5192	-0.0938	0.6781	-0.2124	0.3554
ARA	5,6-EET	0.1056	0.5928	-0.1314	0.5050	-0.1087	0.5818	-0.1104	0.5761	-0.2526	0.1946	-0.3921	0.0391 *	0.1766	0.3686	0.0249	0.9020	0.1031	0.6089	-0.1579	0.4828	-0.2520	0.2705
ARA	ARA	0.5825	0.0011 *	-0.1346	0.4948	-0.3157	0.1018	-0.2425	0.2137	-0.1900	0.3329	-0.3505	0.0674	-0.0180	0.9275	-0.4425	0.0208 *	-0.4863	0.0101 *	-0.2626	0.2376	-0.3070	0.1759

Correlations between ARA metabolites and parameters (P < 0.05). P value by Pearson's correlation coefficient.

Table S8. Correlation between all of DHA metabolites and all of parameters at 4 weeks after nephrectomy

Category	Metabolites	Renal function				Oxidative stress						Inflammation and fibrosis											
		Ccr		Urinary albumin		ROS		ONOO-		IS		TNF-α		TGF-β_1		MT		α-SMA		CD86		CD163	
		r	*P*	*r*	*P*	*r*	*P*	*r*	*P*	*r*	*P*	*r*	*P*	*r*	*P*	*r*	*P*	*r*	*P*	*r*	*P*	*r*	*P*
DHA	Resolvin D3	0.0000	1.0000	0.0000	1.0000	0.0000	1.0000	0.0000	1.0000	0.0000	1.0000	0.0000	1.0000	0.0000	1.0000	0.0000	1.0000	0.0000	1.0000	0.0000	1.0000	0.0000	1.0000
DHA	Resolvin D2	0.0000	1.0000	0.0000	1.0000	0.0000	1.0000	0.0000	1.0000	0.0000	1.0000	0.0000	1.0000	0.0000	1.0000	0.0000	1.0000	0.0000	1.0000	0.0000	1.0000	0.0000	1.0000
DHA	Resolvin D1	0.0000	1.0000	0.0000	1.0000	0.0000	1.0000	0.0000	1.0000	0.0000	1.0000	0.0000	1.0000	0.0000	1.0000	0.0000	1.0000	0.0000	1.0000	0.0000	1.0000	0.0000	1.0000
DHA	Resolvin D4	0.0000	1.0000	0.0000	1.0000	0.0000	1.0000	0.0000	1.0000	0.0000	1.0000	0.0000	1.0000	0.0000	1.0000	0.0000	1.0000	0.0000	1.0000	0.0000	1.0000	0.0000	1.0000
DHA	Maresin1	-0.0402	0.8392	-0.1175	0.5515	0.0371	0.8514	0.0143	0.9424	-0.1046	0.5962	-0.1950	0.3200	-0.0947	0.6318	0.2600	0.1903	0.1057	0.5999	-0.0339	0.8810	-0.0451	0.8462
DHA	10,17-DiHDHA	0.0482	0.8074	0.1834	0.3503	-0.2500	0.1994	-0.1962	0.3171	0.1155	0.5582	-0.1144	0.5621	0.0052	0.9791	-0.4201	0.0291 *	-0.3804	0.0503	-0.2327	0.2974	-0.1560	0.4996
DHA	Resolvin D5	0.2976	0.1240	-0.1684	0.3918	0.0514	0.7950	-0.0997	0.6138	-0.1922	0.3272	-0.2985	0.1229	0.0570	0.7734	-0.1206	0.5492	-0.1664	0.4069	-0.5156	0.0141 *	-0.3568	0.1123
DHA	7,17-hydroxy-DPA	-0.0165	0.9335	-0.0725	0.7138	-0.0558	0.7781	0.0027	0.9889	-0.1000	0.6125	-0.0831	0.6743	-0.0459	0.8164	-0.0052	0.9795	-0.1314	0.5135	0.0953	0.6732	-0.0509	0.8267
DHA	19,20-DiHDPA	0.3083	0.1105	-0.3464	0.0709	-0.1519	0.4403	-0.3557	0.0632	-0.4599	0.0138 *	-0.5054	0.0061 *	0.4105	0.0300 *	-0.2399	0.2281	-0.2841	0.1509	-0.5616	0.0065 *	-0.3941	0.0771
DHA	20-HDHA	0.4478	0.0169 *	-0.4375	0.0199 *	-0.1851	0.3456	-0.3680	0.0540	-0.5151	0.0050 *	-0.5378	0.0032 *	0.3628	0.0578	-0.4749	0.0123 *	-0.4893	0.0096 *	-0.7514	<.0001 *	-0.5324	0.0130 *
DHA	16-HDHA	0.4443	0.0178 *	-0.4138	0.0286 *	-0.1500	0.4461	-0.2903	0.1339	-0.4911	0.0080 *	-0.4595	0.0139 *	0.3119	0.1062	-0.4622	0.0152 *	-0.4745	0.0124 *	-0.7695	<.0001 *	-0.5039	0.0199 *
DHA	17-HDHA	0.0595	0.7636	-0.1536	0.4352	0.1162	0.5559	-0.0818	0.6791	-0.1426	0.4690	-0.1933	0.3244	0.0278	0.8883	-0.0597	0.7676	-0.1995	0.3184	-0.3722	0.0881 *	-0.2512	0.2720
DHA	13-HDHA	0.2066	0.2915	-0.1460	0.4585	0.1256	0.5241	-0.1204	0.5418	-0.2498	0.1999	-0.2106	0.2820	-0.0132	0.9467	-0.1117	0.5792	-0.2998	0.1286	-0.4826	0.0229 *	-0.2600	0.2549
DHA	10-HDHA	0.3683	0.0538	-0.3151	0.1024	-0.1095	0.5790	-0.3261	0.0903	-0.4035	0.0332 *	-0.4885	0.0083 *	0.2748	0.1570	-0.3790	0.0512	-0.4390	0.0220 *	-0.7482	<.0001 *	-0.5210	0.0154 *
DHA	14-HDHA	0.1511	0.4427	0.1092	0.5801	0.0849	0.6677	-0.1892	0.3348	0.0862	0.6627	-0.2968	0.1251	-0.0344	0.8622	-0.1706	0.3948	-0.2145	0.2827	-0.4131	0.0560	-0.3748	0.0942
DHA	11-HDHA	0.3838	0.0438 *	-0.3068	0.1123	-0.1102	0.5766	-0.3343	0.0820	-0.3827	0.0444 *	-0.4307	0.0221 *	0.3128	0.1051	-0.3911	0.0437 *	-0.3968	0.0405 *	-0.7490	<.0001 *	-0.5188	0.0160 *
DHA	7-HDHA	0.3945	0.0377 *	-0.4103	0.0301 *	-0.1027	0.6030	-0.3335	0.0828	-0.4972	0.0071 *	-0.4323	0.0216 *	0.2227	0.2546	-0.4245	0.0273 *	-0.4920	0.0091 *	-0.6641	0.0008 *	-0.4118	0.0636
DHA	8-HDHA	0.3295	0.0868	-0.1864	0.3421	-0.1356	0.4915	-0.4494	0.0164 *	-0.3136	0.1042	-0.5757	0.0013 *	0.2227	0.2547	-0.3736	0.0549	-0.3430	0.0798	-0.6588	0.0009 *	-0.5877	0.0051 *
DHA	17-HpDHA	0.0000	1.0000	0.0000	1.0000	0.0000	1.0000	0.0000	1.0000	0.0000	1.0000	0.0000	1.0000	0.0000	1.0000	0.0000	1.0000	0.0000	1.0000	0.0000	1.0000	0.0000	1.0000
DHA	4-HDHA	0.5826	0.0011 *	-0.4445	0.0178 *	-0.1562	0.4272	-0.4314	0.0219 *	-0.5316	0.0036 *	-0.5825	0.0011 *	0.3171	0.1002	-0.5184	0.0056 *	-0.5613	0.0023 *	-0.8325	<.0001 *	-0.6022	0.0039 *
DHA	19,20-EpDPA	-0.1164	0.5553	-0.0722	0.7150	-0.1046	0.5962	-0.0804	0.6842	-0.1880	0.3381	-0.2299	0.2391	0.2284	0.2423	-0.0149	0.9412	-0.0146	0.9424	-0.1988	0.3752	-0.1373	0.5529
DHA	16,17-EpDPA	-0.0926	0.6392	-0.0228	0.9082	-0.1387	0.4814	-0.0119	0.9521	-0.1543	0.4330	-0.1608	0.4137	0.2606	0.1805	-0.0394	0.8453	-0.0253	0.9003	-0.1796	0.4239	-0.0862	0.7103
DHA	DHA	0.1039	0.5989	0.0449	0.8204	-0.1945	0.3212	-0.1363	0.4890	-0.0749	0.7047	-0.2103	0.2827	0.0336	0.8654	-0.3228	0.1006	-0.4441	0.0203 *	-0.2367	0.2889	-0.1849	0.4224

Correlations between DHA metabolites and parameters ($P < 0.05$). P value by Pearson's correlation coefficient.

Table S9. Correlation between all of EPA metabolites and all of parameters at 4 weeks after nephrectomy

Category	Metabolites	Renal function				Oxidative stress						Inflammation and fibrosis											
		Ccr		Urinary albumin		ROS		ONOO-		IS		TNF-α		TGF-β1		MT		α-SMA		CD86		CD163	
		r	*P*	*r*	*P*	*r*	*P*	*r*	*P*	*r*	*P*	*r*	*P*	*r*	*P*	*r*	*P*	*r*	*P*	*r*	*P*	*r*	*P*
EPA	delta17-6-keto-PGF1α	-0.5311	0.0036 *	0.2765	0.1543	0.2050	0.2953	0.7380	<.0001 *	0.3664	0.0552	0.6113	0.0005 *	-0.2296	0.2398	0.4764	0.0120 *	0.2282	0.2522	0.5703	0.0056 *	0.7907	<.0001 *
EPA	Resolvin E1	0.0000	1.0000	0.0000	1.0000	0.0000	1.0000	0.0000	1.0000	0.0000	1.0000	0.0000	1.0000	0.0000	1.0000	0.0000	1.0000	0.0000	1.0000	0.0000	1.0000	0.0000	1.0000
EPA	8-iso-PGF3α	0.0153	0.9383	0.0345	0.8618	-0.1955	0.3187	0.1014	0.6077	-0.0675	0.7330	0.0011	0.9954	0.2808	0.1477	-0.2543	0.2005	-0.0897	0.6562	-0.1026	0.6496	-0.0707	0.7606
EPA	TXB3	-0.4807	0.0096 *	0.1000	0.6126	0.4326	0.0215 *	0.7497	<.0001 *	0.1816	0.3549	0.6077	0.0006 *	-0.2551	0.1901	0.5960	0.0010 *	0.4223	0.0282 *	0.6507	0.0010 *	0.8914	<.0001 *
EPA	PGF3α	0.0000	1.0000	0.0000	1.0000	0.0000	1.0000	0.0000	1.0000	0.0000	1.0000	0.0000	1.0000	0.0000	1.0000	0.0000	1.0000	0.0000	1.0000	0.0000	1.0000	0.0000	1.0000
EPA	11-dehydro-TXB3	0.0000	1.0000	0.0000	1.0000	0.0000	1.0000	0.0000	1.0000	0.0000	1.0000	0.0000	1.0000	0.0000	1.0000	0.0000	1.0000	0.0000	1.0000	0.0000	1.0000	0.0000	1.0000
EPA	PGE3	-0.0323	0.8706	-0.2744	0.1576	0.1118	0.5711	0.4466	0.0172 *	-0.1267	0.5206	0.0354	0.8579	-0.0312	0.8748	0.1990	0.3197	-0.0859	0.6702	-0.1672	0.4571	0.4578	0.0369 *
EPA	PGD3	0.0463	0.8150	-0.2736	0.1589	-0.0892	0.6519	-0.2142	0.2737 *	-0.3327	0.0837	-0.2846	0.1422	0.1295	0.5113	-0.2844	0.1504	-0.3049	0.1221	-0.4184	0.0527	-0.2235	0.3302
EPA	LXA5	-0.0688	0.7279	-0.1212	0.5391	-0.0967	0.6245	-0.1724	0.3803	-0.1172	0.5527	-0.0576	0.7709	-0.0141	0.9432	-0.2201	0.2700	-0.2436	0.2207	-0.1597	0.4778	-0.0604	0.7948
EPA	LTB5	0.0000	1.0000	0.0000	1.0000	0.0000	1.0000	0.0000	1.0000	0.0000	1.0000	0.0000	1.0000	0.0000	1.0000	0.0000	1.0000	0.0000	1.0000	0.0000	1.0000	0.0000	1.0000
EPA	17,18-DiHETE	0.1322	0.5024	-0.3084	0.1104	-0.1925	0.3263	-0.2794	0.1498	-0.4017	0.0341 *	-0.3656	0.0557	0.4566	0.0146 *	-0.2692	0.1745	-0.2249	0.2595	-0.4548	0.0334 *	-0.2539	0.2668
EPA	14,15-DiHETE	-0.0645	0.7442	-0.1499	0.4465	-0.1016	0.6068	-0.2028	0.3005	-0.2608	0.1801	-0.2414	0.2159	0.3485	0.0692	-0.1255	0.5328	-0.0651	0.7469	-0.3070	0.1646	-0.1927	0.4028
EPA	5,6-DiHETE	0.1855	0.3447	-0.2556	0.1892	-0.1046	0.5963	-0.2868	0.1390	-0.2834	0.1439	-0.3281	0.0883	0.2706	0.1636	-0.1962	0.3267	-0.2530	0.2030	-0.3055	0.1668	-0.1912	0.4065
EPA	18-HEPE	0.3612	0.0589	-0.4424	0.0184 *	-0.2624	0.1774	-0.3582	0.0612	-0.5367	0.0032 *	-0.4803	0.0097 *	0.4156	0.0278 *	-0.5323	0.0043 *	-0.4911	0.0093 *	-0.6221	0.0020 *	-0.4124	0.0632
EPA	15-HEPE	0.1591	0.4187	-0.3296	0.0867	-0.0346	0.8613	-0.2434	0.2120	-0.2945	0.1282	-0.2532	0.1936	0.2378	0.2230	-0.2309	0.2466	-0.3272	0.0958	-0.4574	0.0323	-0.1894	0.4109
EPA	11-HEPE	0.0053	0.9787	-0.3189	0.0982	-0.0368	0.8524	0.0919	0.6420	-0.3167	0.1006	-0.1454	0.4602	0.1071	0.5876	-0.0657	0.7448	-0.2948	0.1355	-0.3452	0.1156	0.2269	0.3226
EPA	8-HEPE	0.4843	0.0090	-0.2450	0.2088	-0.1914	0.3292	-0.1955	0.3188	-0.2535	0.1931	-0.2018	0.3032	0.2169	0.2675	-0.2956	0.1344	-0.3334	0.0892	-0.3093	0.1614	-0.1760	0.4453
EPA	9-HEPE	0.0000	1.0000	0.0000	1.0000	0.0000	1.0000	0.0000	1.0000	0.0000	1.0000	0.0000	1.0000	0.0000	1.0000	0.0000	1.0000	0.0000	1.0000	0.0000	1.0000	0.0000	1.0000
EPA	12-HEPE	0.1234	0.5316	-0.2263	0.2469	-0.0832	0.6738	-0.2843	0.1425	-0.2756	0.1558	-0.2809	0.1476	0.2476	0.2041	-0.2961	0.1338	-0.3092	0.1165	-0.5542	0.0074 *	-0.3489	0.1211
EPA	5-HEPE	0.2203	0.2600	-0.3932	0.0385 *	-0.2482	0.2028	-0.4147	0.0282 *	-0.4834	0.0092 *	-0.4032	0.0334 *	0.3590	0.0606	-0.5244	0.0050 *	-0.4662	0.0142 *	-0.5768	0.0049 *	-0.3671	0.1017
EPA	15-HpEPE	0.0000	1.0000	0.0000	1.0000	0.0000	1.0000	0.0000	1.0000	0.0000	1.0000	0.0000	1.0000	0.0000	1.0000	0.0000	1.0000	0.0000	1.0000	0.0000	1.0000	0.0000	1.0000
EPA	12-HpEPE	0.0000	1.0000	0.0000	1.0000	0.0000	1.0000	0.0000	1.0000	0.0000	1.0000	0.0000	1.0000	0.0000	1.0000	0.0000	1.0000	0.0000	1.0000	0.0000	1.0000	0.0000	1.0000
EPA	5-HpEPE	0.0000	1.0000	0.0000	1.0000	0.0000	1.0000	0.0000	1.0000	0.0000	1.0000	0.0000	1.0000	0.0000	1.0000	0.0000	1.0000	0.0000	1.0000	0.0000	1.0000	0.0000	1.0000
EPA	17,18-EpETE	-0.1821	0.3537	-0.0853	0.6661	-0.1095	0.5791	-0.0110	0.9558	-0.2015	0.3037	-0.0912	0.6445	0.3162	0.1011	-0.0683	0.7351	0.0838	0.6777	-0.1940	0.3870	-0.0036	0.9876
EPA	14,15-EpETE	-0.1314	0.5049	-0.0922	0.6408	-0.1361	0.4898	-0.1038	0.5991	-0.2113	0.2805	-0.1657	0.3993	0.3191	0.0979	-0.1281	0.5243	-0.0049	0.9806	-0.2195	0.3265	-0.1275	0.5818
EPA	EPA	0.0130	0.9476	-0.1282	0.5156	-0.2161	0.2694	-0.1366	0.4881	-0.2235	0.2530	-0.1552	0.4304	0.2442	0.2105	-0.3885	0.0452	-0.3403	0.0824	-0.3271	0.1373	-0.0666	0.7743

Correlations between EPA metabolites and parameters ($P < 0.05$). P value by Pearson's correlation coefficient.
